# Risk assessment requires several bee species to address species-specific sensitivity to insecticides at field-realistic concentrations

**DOI:** 10.1038/s41598-023-48818-7

**Published:** 2023-12-18

**Authors:** Tobias Jütte, Anna Wernecke, Felix Klaus, Jens Pistorius, Anke C. Dietzsch

**Affiliations:** Institute for Bee Protection, Julius Kuehn-Institute (JKI), Federal Research Centre for Cultivated Plants, Messeweg 11-12, 38104 Braunschweig, Germany

**Keywords:** Agroecology, Biodiversity, Conservation biology, Ecosystem services, Ecology

## Abstract

In the European registration process, pesticides are currently mainly tested on the honey bee. Since sensitivity data for other bee species are lacking for the majority of xenobiotics, it is unclear if and to which extent this model species can adequately serve as surrogate for all wild bees. Here, we investigated the effects of field-realistic contact exposure to a pyrethroid insecticide, containing lambda-cyhalothrin, on seven bee species (*Andrena vaga*, *Bombus terrestris*, *Colletes cunicularius*, *Osmia bicornis*, *Osmia cornuta, Megachile rotundata, Apis mellifera*) with different life history characteristics in a series of laboratory trials over two years. Our results on sensitivity showed significant species-specific responses to the pesticide at a field-realistic application rate (i.e., 7.5 g a.s./ha). Species did not group into distinct classes of high and low mortality. Bumble bee and mason bee survival was the least affected by the insecticide, and *M. rotundata* survival was the most affected with all individuals dead 48 h after application. *Apis mellifera* showed medium mortality compared to the other bee species. Most sublethal effects, i.e. behavioral abnormalities, were observed within the first hours after application. In some of the solitary species, for example *O. bicornis* and *A. vaga,* a higher percentage of individuals performed some abnormal behavior for longer until the end of the observation period. While individual bee weight explained some of the observed mortality patterns, differences are likely linked to additional ecological, phylogenetic or toxicogenomic parameters as well. Our results support the idea that honey bee data can be substitute for some bee species’ sensitivity and may justify the usage of safety factors. To adequately cover more sensitive species*,* a larger set of bee species should be considered for risk assessment.

## Introduction

In recent years, the decline in global biodiversity, particularly the decline in bees^1–3^, is an ongoing topic of public and scientific discussions. As one of its proposed drivers, conventional agriculture and the use of pesticides have generated substantial debate^4,5^. In this context, the comprehensiveness of the process and scientific basis for the approval of pesticides in the EU have been questioned, since it only considers primarily the honey bee (*Apis mellifera* L.) for risk assessment of pesticides^6,7^.

Not only for ecosystem functioning but also for ecosystem services such as crop pollination, wild bee species are of enormous importance^8–10^. They show numerous differences in their life history traits compared to the honey bee and, due to their potentially higher ecological and toxicological sensitivity, may be exposed to additional risks from pesticides that may or may not be covered by risk assessment procedures developed for honey bees^11–13^. The life history traits of wild bees induce further exposure pathways, such as exposure via nesting location or via nesting substrates for brood cells (e.g. soil and leaf material), affecting both adult bees and juvenile stages (for a more comprehensive review of differences in pathways compare^14^). These pathways are irrelevant for the honey bee that is mainly exposed to pesticides via pollen, nectar, and to a lower extent, guttation water^6^. Bee susceptibility to other stressors, such as parasites, pathogens, shortage of foraging plants, and nesting habitats, is modified by bee characteristics. Important characteristics are for example the size or weight of a bee individual, which is known to positively correlate with flight radius, bee behavior (e.g., nest usurpation behavior) and fitness^15–17^. Body size and detoxification capacity of bees are also crucial for their responses to pesticide exposure^18,19^.

Most data on sensitivity, often measured as a lethal (mortality) and a sublethal (abnormal behavior) component, are only available for a few wild bee species (e.g., *Bombus terrestris*, *Osmia bicornis*; cf. review^14^), and come with limitations (methodologies of different studies are not comparable, non-standardized sampling times), so that it is difficult to compare species across different studies^20^. To address these shortcomings, we carried out comparative trials on contact exposure of seven social and solitary bee species with the pyrethroid lambda-cyhalothrin, including the honey bee as a reference. Pesticides with the active substance lambda-cyhalothrin are commonly used as applications in bee-attractive crops such as apples or oilseed rape^21,22^, where they may be sprayed during flowering time of the crops and flight time of bees. Laboratory studies (i.e., worst case scenarios) demonstrated its toxicity in honey bees via contact exposure, yet no adverse toxicity effects were revealed under semi-field or field conditions^23^. Such a toxicity profile for honey bees was a precondition for the choice of an active substance in our experiment in order to classify sensitivity in other bee species that is yet unknown. We define sensitivity as the mortality of and sublethal effects to individual bees of a species over time. Hence, sensitivity is not synonymous with vulnerability, which also includes the likelihood of exposure^24^, but it is rather an aspect of the probability of vulnerability.

## Material and methods

### Model organisms

**Figure 1:**
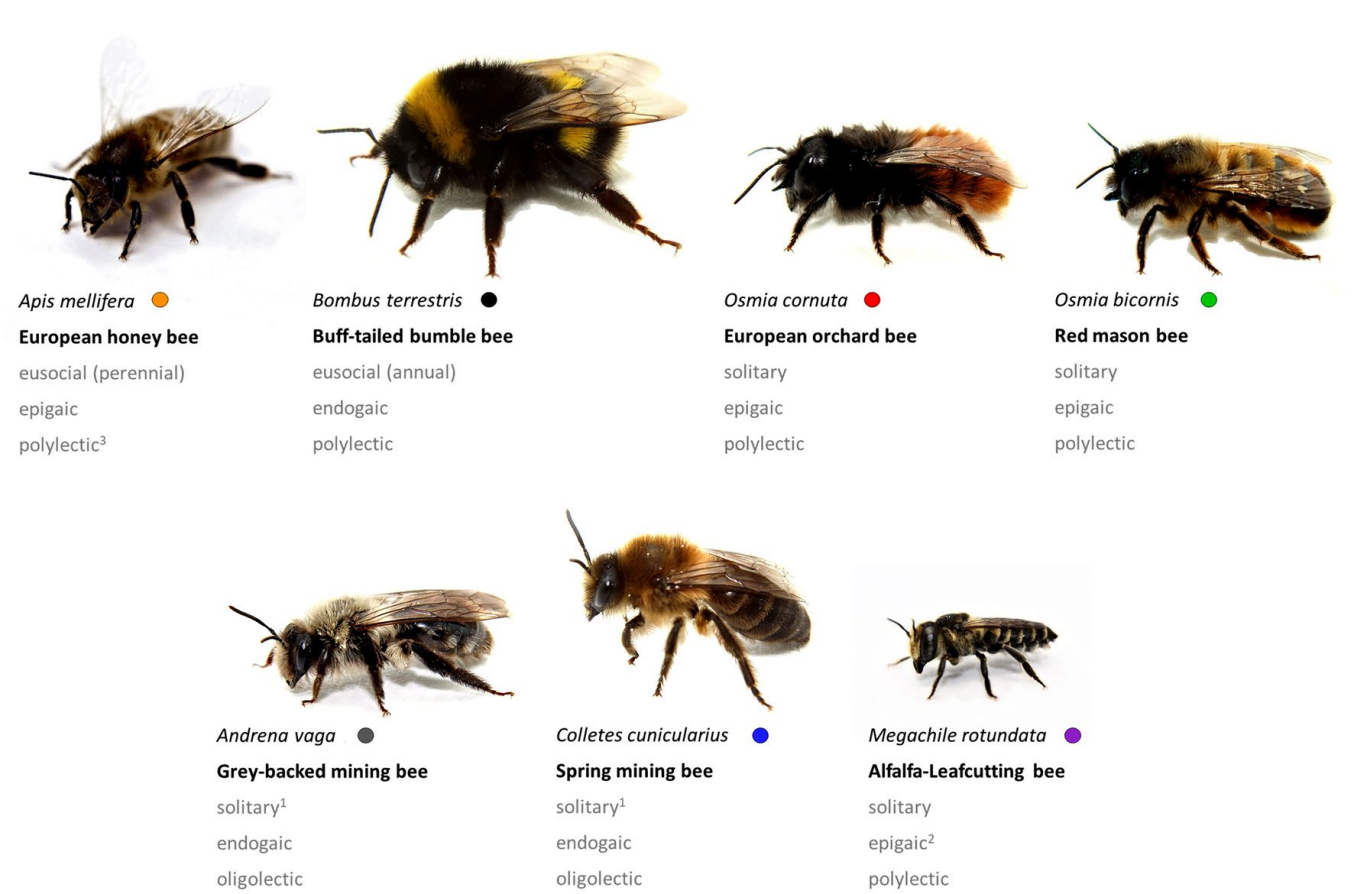
Scientific and common names of bee species used in the trials and some important traits of their life history in consecutive order: sociality (eusocial = form of social organization, involving a reproductive division of labor among members, overlapping generations, and cooperative care of juvenile stages, solitary = live independently as reproductive unit), nest location (epigaic = above ground, endogaic = below ground) and food preference (oligolectic = specialization or preference for a defined pollen source, polylectic = collection of pollen from a variety of sources). ^1^species forms nest aggregations; ^2^species uses leaf material of few plant species for brood cells; ^3^species often uses mass flowering crops.

Two social bee species, *Apis mellifera* and *Bombus terrestris*, and five solitary bee species, *Osmia bicornis*, *Osmia cornuta*, *Andrena vaga*, *Colletes cunicularius*, and *Megachile rotundata*, were used to cover various characteristics and life history traits (Fig. 1). All species are native to Central Europe and have been shown to pollinate both crop and wild plant species^25,26^.

Honey bee individuals were sampled from five queen-right colonies at the Institute for Bee Protection, Braunschweig, Germany. The queens descend from a breeding line reared in the same year at the test facility. Bees were taken from near the brood nest to standardize the age of the bees. *Bombus terrestris* individuals were sampled from commercial hives ordered from BioBest (Westerlo, Belgien). *Osmia bicornis* and *O. cornuta* individuals were delivered by WAB Mauerbienenzucht (Konstanz, Germany) and Bienenhotel.de (Neuenkirchen, Germany). *Megachile rotundata* bee individuals were imported from Northstar Seed (Okotoks, Alberta, Canada). Individuals of the other two solitary bee species were caught at different locations in Braunschweig during a period of 10 days before the start of the trials, hence we were not able to standardize their exact individual ages as we did for the commercially available species (Supplement 1). In total, we tested 409 *A. mellifera*, 385 *B. terrestris*, 383 *O. bicornis*, 139 *O. cornuta*, 140 *M. rotundata*, 105 *A. vaga,* and 103 *C. cunicularius* individuals in six trials (Supplement 2). We exclusively used female individuals as their active foraging and effects of exposure to pesticides are directly (or in the case of worker bees indirectly) linked to reproduction^14^.

### Experimental design

For the experiment, trials were run in 2018 (April–August) and 2019 (March–July, Supplement 2). Species caught in the wild were tested in fewer trials than commercially available species due to few temporal opportunities to catch them (Supplement 2). The experimental approach imitated a contact exposure of lambda-cyhalothrin (Karate® Zeon) at a field-realistic application rate of 0.075 L product/ha, i.e., 7.5 g a.s./ha (corresponding to the maximum authorized application rate in oil seed rape). We used a professional spray chamber (custom-built by Christan Schachtner Gerätetechnik, Ludwigsburg, Germany) rather than the standard topical application. This chamber mimics a field-realistic application scenario and exposure, in which bees may fly through insecticide spray applied by field sprayers when they pass through or nest^27^ in a field or when insecticide spray drifts to field-adjacent areas (flowering strips, hedges) that are used by bees for foraging and/or nesting. The method was implemented as described in references^28,29^ with minor modifications (cf. Supplement 3). Tap water was used as a control. Dimethoate (Dandadim^©^ Progress) was used at an application rate of 1.0 L product/ha, i.e., 400 g a.s./ha as a toxic reference in each trial to serve as an indicator for a valid test system^30,31^. We did not include it in all statistical analyses and hence used a smaller number of replicates (one or two cages for honey bee trials and trials for each other species, respectively; Supplement 2). Experimental implementation was in accordance with the Guidelines for the Testing of Chemicals 214^30^ and 246^31^.

The day before application, bees were assigned to standard stainless steel cages (10 cm × 8.5 cm × 5.5 cm) with only bee individuals of the same species sharing a cage. Assignment was conducted in a way that the size of individuals and average bee weight per cage (deviation ≤ 12%; Supplement 2) was comparable between cages of the same species, i.e., cages were true replicates. Before and during the trials, cages of all species were kept in a climate chamber at an average temperature of 24 °C ± 1 °C (resembling field-realistic temperatures) and an average relative humidity of 60% ± 10% in darkness. Bees were fed ad libitum with 50–55% sugar solution (w/v). Cages were then assigned to the treatment, control or toxic reference group.

On the day of application bee individuals were immobilized by cooling them down before transferring all bee individuals from one cage to a petri dish and spraying them at room temperature with the treatment, control or toxic reference solution (spray speed: 2.5 km/h; spray height: 42 cm; nozzle pressure: 2.9 bar; system pressure: 7–8 bar; setting: 300 l water/ha; nozzle type: Teejet 9503 EVS, commercially available). Immediately after the application, bees were transferred back into their cage and kept for further observation in the climate chamber. Individual bee mortality and behavior of bee individuals that were still alive were visually checked at pre-defined time intervals (2, 4, 24, 48, 72 and 96 h). In line with OECD guidelines^32^, observed behavioral abnormalities were categorized (moribund, cramps, apathy, affected [including restless, vertigo, uncoordinated and dorsal position] and symptoms [including all of the above except for apathy; see below]). Control mortality 96 h after application was ≤ 10% in each of the trials for each bee species except for one trial with *A. vaga* (17%), one trial with *C. cunicularius* (13%) and one trial with *O. bicornis* (13%). While the average control mortality across all *A. vaga* and all *O. bicornis* trials did not exceed 10% respectively as required by OECD guidelines^30^, the average control mortality across all *C. cunicularius* trials was 11%. This slightly higher than recommended control mortality was considered valid in light of ICPPR test protocols suggesting a change of control mortality to 15–20% in accordance to other non-target arthropod testing^33,34^. We included all control data in further analyses. In the toxic reference, cumulative mortality exceeded 50% for all bee species, except for one bumble bee trial in 2019. In this trial all other bee species treated with the identical toxic reference solution showed 100% mortality after 96 h.

### Statistical analysis

#### Mortality

Survival probability of individual bees (= 1-mortality) over time was analyzed using a full mixed effects Cox model^35^. In contrast to the Kaplan–Meier method, mixed effects Cox models can adjust for confounding effects by adding random variables and can quantify the difference in survival between groups by providing effect estimates^36^. We conducted our analyses with an unbalanced dataset due to commercially available species being used in more trials than bee species caught in the wild; this is mirrored by larger confidence intervals for the less often used species. The model included *species* (seven levels: *A. mellifera, B. terrestris, O. bicornis, O. cornuta, M. rotundata, A. vaga,* and *C. cunicularius*), *treatment* (two levels: lambda-cyhalothrin = T, control = C) and their interaction as fixed factors and *cage number* nested within *trial number* as random factors. *trial number* refers to each conducted trial as stated in Supplement 2 (“trial no.”, N = 6), while *cage number* is a unique number for each individual cage used across all trials, species and treatments (N = 251, cf. Supplement 2). The final model was selected by comparing full and reduced models (using anova.coxme^35^) and excluding non-significant components (Supplement 4); the final model contained *species* and *treatment*, and their interaction, and *cage number* as a random effect. With post hoc tests, we compared survival probability of treated and control bees within each species as well as survival probability of treated bees between species using multivariate t-distribution adjustment (mvt) to account for multiple testing^37^ (cf. also^38^). We used the same workflow for analyzing dimethoate contact exposure.

We did not conduct any model diagnostic test or plotting, since diagnostic tools are currently not available for mixed effects Cox models^39^. Since the Cox method provides a simulated survival graph, survival curves were plotted using the Kaplan–Meier method^36^. All statistical analyses and plots were performed in R (version 4.2.2^40^) using packages survival (version 3.4-0^41^), coxme (version 2.2-18.1^35^), emmeans (version 1.8.2^42^ for post-hoc tests and mean hazard rates) and ggplot2 (version 3.3.6^43^). We considered results with *p* < 0.05 to be statistically significant.

#### Sublethal effects

The probability of observing abnormal behavior of different categories (moribund, cramps, apathetic, affected, and symptoms [which included all four previous categories]) in all bee individuals alive were first visualized with a heatmap over time using the R package ggplot2 (version 3.3.6^43^). Since not only treatment bees but also control bees displayed apathetic behavior (Fig. 3), this category was excluded from the category “symptoms” and from further analyses. For all other parameters, control bees showed an absence or very low percentages of bees with the behavior in all species (Fig. 3, Table 4). We then modelled behavioral responses of the remaining four categories in lambda-cyhalothrin-treated bees using binomial Generalized Linear Mixed Models (GLMM) with and without quadratic terms and with a logit link. Full models included bee *species* (seven levels), sampling *time* (continuous) and their interaction as explanatory variables, and *cage number* nested within *trial number* as random factors. If model diagnostics (run with the R-package DHARMa, version 0.4.6^44^) indicated dispersion or inflation problems, we refitted models with a zero-inflation term and/or binomial Generalized Additive Mixed Models (GAMM) with a logit link. We used Akaike information criterion (AIC) and Log-Likelihood for model selection; models with the lowest AIC that passed model diagnostics were considered as the final models (Supplement 10). To estimate differences in abnormal behavior between bee species, we conducted post hoc tests at the beginning (2 h), middle (24 h, 48 h), and end (96 h) of the sampling *time* using mvt to account for multiple testing^37^. Statistical analyses and plotting were performed with the R packages glmmTMB (version 1.1.7^45^), gamm4 (version 0.2-6^46^), emmeans (version 1.8.2^42^), ggplot2 (version 3.3.6^43^), gridExtra (version 2.3^47^), multcomp (version 1.4-25^48^) and ggpubr (version 0.6.0^49^).

In the result section, means and standard errors are stated as mean ± SE, confidence interval is abbreviated as CI.

## Results

### Mortality

Exposure to dimethoate resulted in a mean bee mortality of 84 ± 54.1% SE 24 h after application (95 ± 5.0% SE 48 h after application) indicating a sufficient reliability of the experimental setup (cf. also Supplement 2). Estimated mean hazard rates (HZ) ± SE in the dimethoate treatment were 0.84 ± 0.32 SE for *A. vaga,* 0.32 ± 0.06 for *B. terrestris,* 0.85 ± 0.32 for *C. cunicularius,* 0.89 ± 0.29 for *M. rotundata,* 1.02 ± 0.17 for *O. bicornis,* and 1.14 ± 0.37 for *O. cornuta* compared to 2.98 ± 0.56 for *A. mellifera*. *Bombus terrestris* individuals were significantly less likely to die from a dimethoate treatment than *A. mellifera*, *O. cornuta* and *O. bicornis* (*p* ≤ 0.03, mvt method) while *A. mellifera* was significantly more likely to die from dimethoate than *B. terrestris*, *M. rotundata* and *O. bicornis* (*p* ≤ 0.05, mvt method).

**Figure 2:**
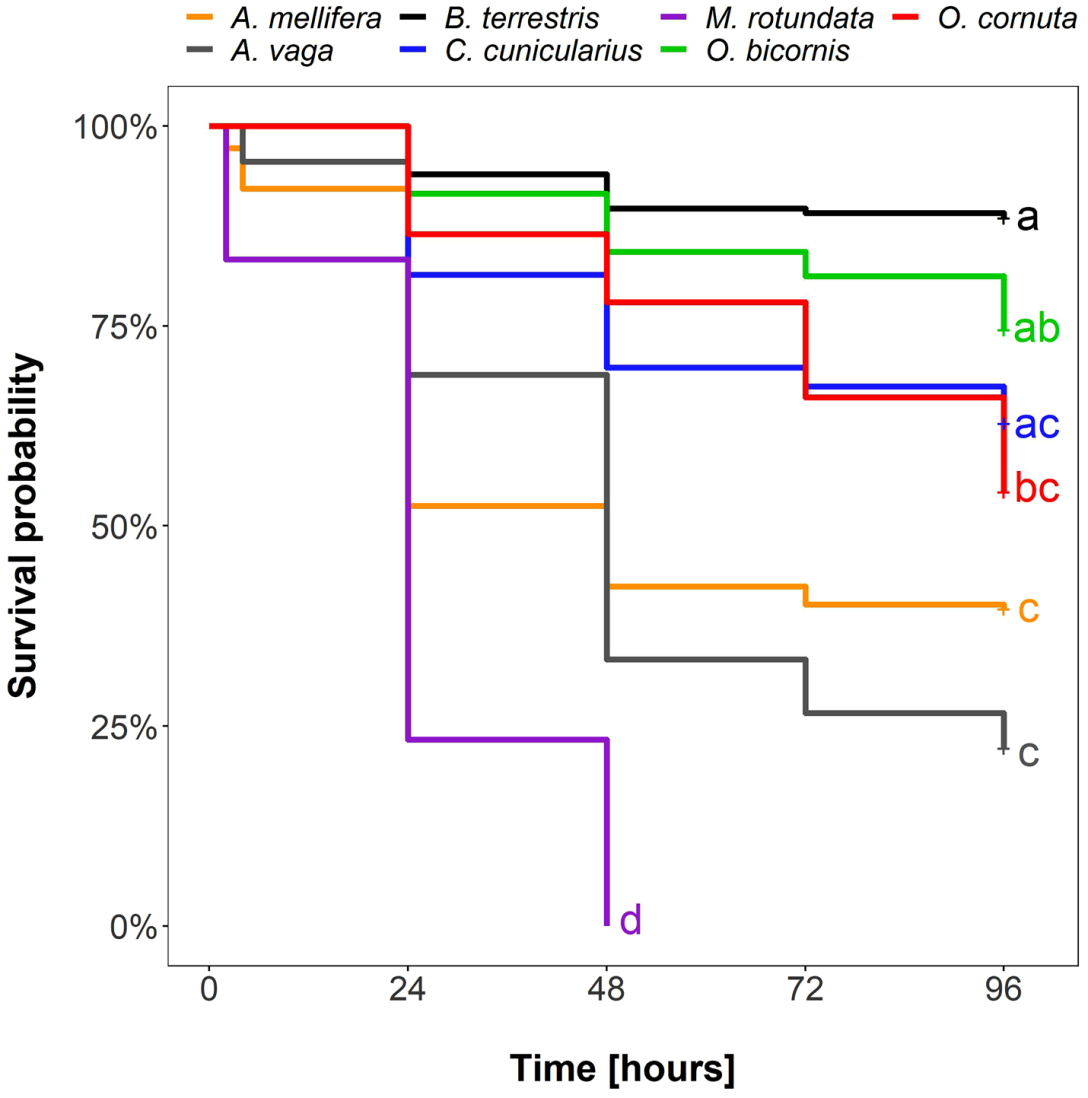
Survival probability (in %) over time (in hours) of individual species treated with lambda-cyhalothrin (Karate® Zeon). A Kaplan–Meier curve is displayed for each species (colored lines). Significant differences (*p* ≤ 0.05, based on post hoc tests of Cox mixed effects model, see Table 2) are identified by different letters.

For each of the seven species, bees in the lambda-cyhalothrin treatment group revealed a significant increase in mortality after insecticide application compared to bees of the control group (z ≤ − 2.1, *p* ≤ 0.035; Table 1, Supplement 5﻿). Hazard of mortality was 17-fold higher among bees in the insecticide treatment group compared to the control group (95% CI 10.8, 26.9). While bee individuals of the control group did not show significant differences in mortality among species (z ≤|− 2.3|, *p* ≥ 0.24 for pairwise comparisons within the control group, Supplement 6), they significantly differed in their response to the lambda-cyhalothrin treatment (Fig. 2, Table 2). *Apis mellifera* showed a significantly higher mortality than *B. terrestris* (ratio 9.97; z = 6.6, *p* < 0.001) and *O. bicornis* (ratio 4.26; z = 4.7, *p* < 0.001), but its mortality was significantly lower than in *M. rotundata* (ratio 0.21, z = 4.7, *p* < 0.001). *Bombus terrestris* individuals responded in their mortality significantly less sensitive to the insecticide treatment than all other species except for *O. bicornis* and *C. cunicularius* (z ≤|− 2.886|, *p* ≥ 0.06; Fig. 2, Table 2). Estimated model hazard rates in the lambda-cyhalothrin group ranged from 0.87 (CI 0.42, 1.84) in *B. terrestris* to 41.14 (CI 18.97, 89.23) in *M. rotundata* (Supplement 7) with *A. mellifera*’s HZ being intermediate (8.7; CI 4.81, 15.81). Mortality rates 96 h after application ranged for the control group from a mean of 1.8 ± 1.0% across trials in *B. terrestris* to 11.1 ± 4.7% in *C. cunicularius* (*A. mellifera* 2.4 ± 1.2%, *O. cornuta* 3.3 ± 2.3%, *O. bicornis* 4.3 ± 1.6%, *M. rotundata* 8.3 ± 3.6%, *A. vaga* 11.1 ± 4.7%). Bees treated with lambda-cyhalothrin showed significantly higher mortality rates than control bees between 11.5 ± 2.5% SE in *B. terrestris* and 100 ± 0% in *M. rotundata* (*O. cornuta* 45.8 ± 6.5%, *O. bicornis* 25.5 ± 3.4%, *C. cunicularius* 37.2 ± 7.5%, and *A. vaga* 77.8 ± 6.3%, *A. mellifera* 60.3 ± 3.7%) 96 h after insecticide application (Fig. 2). Even though some species revealed relatively similar (not-significantly different) mortalities to each other, they did not form a distinct cluster with significantly lower or higher mortality rates (Fig. 2) in comparison to a group of other tested species. The only exception was *M. rotundata,* which revealed a significantly higher mortality than all other species in our experiment (Fig. 2, Table 2).

**Table 1:** Post hoc contrasts of Cox mixed-effects model comparing levels of *treatment* (water = C vs. lambda-cyhalothrin = T) within each bee species (cf. Supplement 5). Results are given on the model scale (log scale); results at a significance level of alpha = 0.05 are highlighted in bold.

Bee species	Contrast	Estimate	Standard error	Z ratio	*p* value
*Apis mellifera*	C versus T	− 3.96	0.585	− 6.777	**< 0.001**
*Andrena vaga*	C versus T	− 2.64	0.639	− 4.130	**< 0.001**
*Bombus terrestris*	C versus T	− 1.87	0.660	− 2.833	**0.005**
*Colletes cunicularius*	C versus T	− 1.40	0.663	− 2.106	**0.035**
*Megachile rotundata*	C versus T	− 4.21	0.585	− 7.205	**< 0.001**
*Osmia bicornis*	C versus T	− 1.85	0.465	− 3.983	**< 0.001**
*Osmia cornuta*	C versus T	− 2.80	0.818	− 3.430	**< 0.001**

**Table 2:** Comparisons of mortality between individual species after treatment with lambda-cyhalothrin.

Bee species contrasts	Estimate	SE	Z ratio	*p* value
*Apis mellifera–Andrena vaga*	− 0.260	0.414	− 0.627	0.996
*Apis mellifera–Bombus terrestris*	2.300	0.348	6.614	**< 0.001**
*Apis mellifera–Colletes cunicularius*	0.916	0.447	2.048	0.377
*Apis mellifera–Megachile rotundata*	− 1.551	0.365	− 4.255	**< 0.001**
*Apis mellifera–Osmia bicornis*	1.449	0.308	4.698	**< 0.001**
*Apis mellifera–Osmia cornuta*	0.768	0.392	1.958	0.434
*Bombus terrestris–Andrena vaga*	− 2.560	0.437	− 5.851	**< 0.001**
*Bombus terrestris–Colletes cunicularius*	− 1.384	0.480	− 2.886	0.058
*Bombus terrestris–Megachile rotundata*	− 3.851	0.385	− 9.994	**< 0.001**
*Bombus terrestris–Osmia bicornis*	− 0.851	0.357	− 2.382	0.201
*Bombus terrestris–Osmia cornuta*	− 1. 532	0.426	− 3.599	**0.006**
*Andrena vaga–Colletes cunicularius*	1.175	0.517	2.273	0.251
*Andrena vaga–Megachile rotundata*	− 1.292	0.439	− 2.943	**0.049**
*Andrena vaga–Osmia bicornis*	1.709	0.405	4.223	**< 0.001**
*Andrena vaga–Osmia cornuta*	1.028	0.469	2.191	0.294
*Colletes cunicularius–Megachile rotundata*	− 2.467	0.472	− 5.232	**< 0.001**
*Colletes cunicularius–Osmia bicornis*	0.533	0.449	1.189	0.896
*Colletes cunicularius–Osmia cornuta*	− 0.148	0.506	− 0.291	1.000
*Megachile rotundata–Osmia bicornis*	3.001	0.346	8.674	**< 0.001**
*Megachile rotundata–Osmia cornuta*	2.320	0.416	5.581	**< 0.001**
*Osmia bicornis–Osmia cornuta*	− 0.681	0.391	− 1.741	0.581

Estimates of contrasts for all *species’* (seven levels) comparisons, their standard errors (SE) and Z ratios are displayed. The post hoc analysis used multivariate t distribution adjustment (mvt) to account for multiple testing. Results are on the log scale, which is used in the Cox mixed effects model. Significant contrasts at a level of alpha = 0.05 are depicted with *p* values in bold. Negative estimates indicate that the first species is less sensitive to the pesticide than the second species in the bee species contrasts column; positive estimates indicate the first species is more sensitive than the second species.

### Sublethal effects

**Figure 3:**
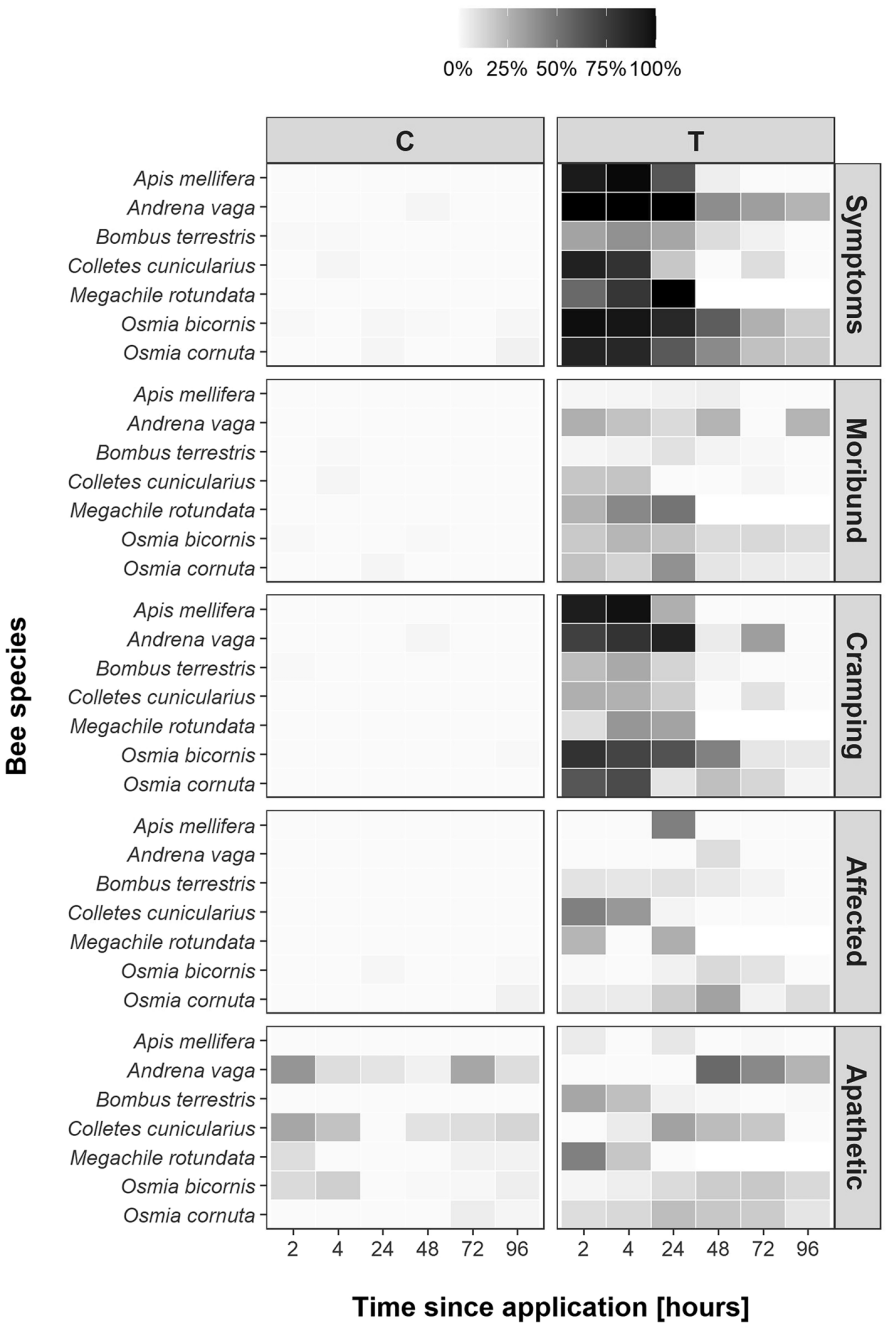
Heatmap of mean recorded abnormal behavior for bees of different species in control cages (C) and bees treated with lambda-cyhalothrin (T) at different sampling times (in hours). A darker shading indicates a higher percentage of bees displaying the particular behavior. Symptoms include the behavioral categories moribund, cramping, and affected.

**Figure 4:**
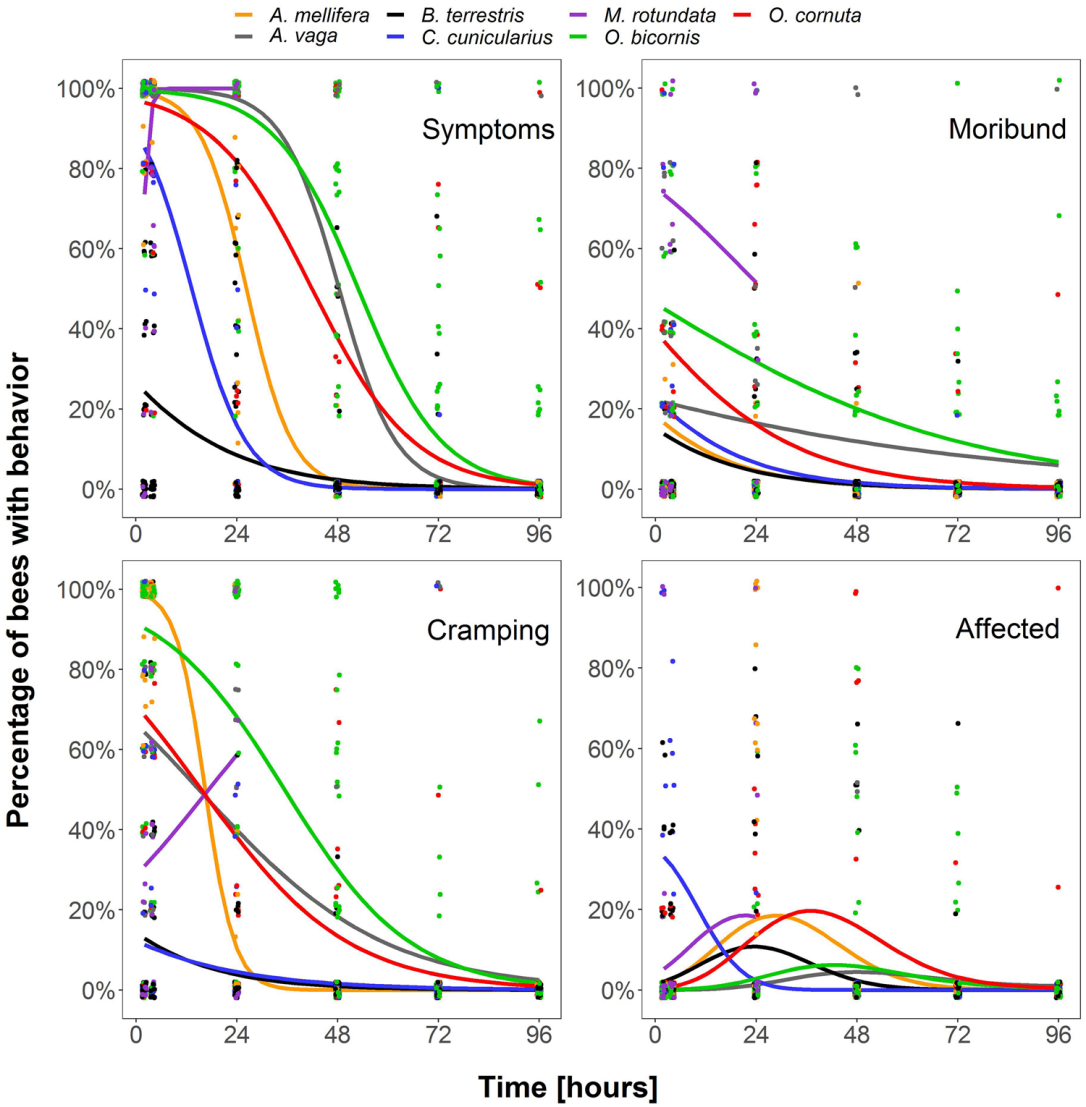
Percentage of observed individuals of tested bee species showing behaviorial abnormalities (i.e., abnormal symptomatic behavior, being moribund, cramping or affected) at different sampling times after application of lambda-cyhalothrin. Solid lines show model estimates for each bee species over time. Filled circles display jittered raw data of observed behavior for each cage, trial and bee species.

Sublethal effects (measured as behavioral abnormalities) did in some cases mirror species-specific mortality. *Bombus terrestris*, which displayed a lower mortality than all other tested species except *C. cunicularius* and *O.bicornis*, also showed a significantly lower proportion of bees with abnormal behavior than all other bee species two as well as 24 h after insecticide application (z ≤ − 3.81, *p* ≤ 0.002; Fig. 3, Table 3) except *C. cunicularius*. In other species with a low mortality sublethal effects did not mirror mortality data. *Osmia bicornis*, which showed significantly lower mortality than three other species, revealed significantly more individuals with symptoms of abnormal behavior two hours after application when compared to *B. terrestris* (z = 11.05, *p* ≤ 0.001, Fig. 3, Table 3), *C. cunicularius* (z = 4.02, *p* = 0.001), and *M. rotundata* (z = 5.09, *p* ≤ 0.001), 24 and 48 h after application compared to *B. terrestris* (z ≥ 7.35, *p* ≤ 0.001), *C. cunicularius* (z ≥ 5.21, *p* ≤ 0.001), and *A. mellifera* (z ≥ 4.60, *p* ≤ 0.001), and even 96 h later when compared to *A. mellifera* and *C. cunicularius* (z ≥ 3.79, *p* ≤ 0.002). Symptoms in *O. bicornis* were mainly related to a higher percentage of moribund and cramping individuals (Fig. 4, Table 4, and Supplement 11) and were observed until the end of the trials (96 h). *Andrena vaga*, whose individuals showed significantly higher mortality than *B. terrestris* and *O. bicornis*, also revealed a significantly greater percentage of individuals with symptoms compared to *B. terrestris* (z = 4.99, *p* < 0.001)*, C. cunicularius* (z = 3.12, *p* = 0.026), and *M. rotundata* (z = 3.36, *p* = 0.012) two hours after application, *B. terrestris* (z = 5.40, *p* < 0.001) and *C. cunicularius* (z = 4.46, *p* < 0.001) 24 h after application, and *B. terrestris* (z = 3.58, *p* < 0.001), *C. cunicularius* (z = 4.08, *p* < 0.001), and *A. mellifera* (z = 3.47, *p* = 0.006) 48 h after application (Table 4). Symptoms in *A. vaga* were mainly related to moribund and cramping individuals (Fig. 4, Table 4, and Supplement 11) and were measurable until the end of the trials. The percentage of honey bees that showed symptoms two hours after application was 94% (Table 4) and significantly higher in comparion to *B. terrestris* (27%; z = 9.15, *p* < 0.001)*, M. rotundata* (51%, z = 4.03, *p* = 0.001), and *C. cunicularius* (92%; z = 3.18, *p* = 0.022) but decreased rapidly to 63% 24 h after application, being only significantly different to the percentage of *B. terrestris* individuals showing symptoms (35%, z = 5.28, *p* < 0.001). Symptoms in honey bees were mainly related to honey bees experiencing cramps two hours (92% of alive individuals) and four hours (98%) after application (Fig. 4, Table 4). At 48 h after application, all other species showed a significantly higher percentage of bees cramping than *A. mellifera* (Table 4).

**Table 3:** Comparison of four categories of abnormal behavior between bee species at four sampling times (2, 24, 48 and 96 h) after treatment with lambda-cyhalothrin.

Behavior	Time	Bee species contrasts	Estimate	SE	Z	*p*
Symptoms	2	*A. mellifera–B. terrestris*	5.60	0.612	9.15	< 0.001
		*A. mellifera–C. cunicularius*	2.70	0.851	3.18	0.022
		*A. mellifera–M. rotundata*	3.43	0.852	4.03	0.001
		*A. vaga–B. terrestris*	8.00	1.603	4.99	< 0.001
		*A. vaga–C. cunicularius*	5.11	1.638	3.12	0.026
		*A. vaga – M. rotundata*	5.84	1.740	3.36	0.012
		*B. terrestris–M. rotundata*	− 2.89	0.759	− 3.81	0.002
		*B. terrestris–O. bicornis*	− 6.26	0.566	− 11.05	< 0.001
		*B. terrestris–O. cornuta*	− 4.46	0.744	− 5.99	< 0.001
		*C. cunicularius–O. bicornis*	− 3.36	0.836	− 4.02	0.001
		*M. rotundata–O. bicornis*	− 4.09	0.804	− 5.09	< 0.001
	24	*A. mellifera–B. terrestris*	2.80	0.530	5.28	< 0.001
		*A. mellifera–M. rotundata*	− 25.45	6.620	− 3.85	0.002
		*A. mellifera–O. bicornis*	− 2.49	0.540	− 4.60	< 0.001
		*A. vaga–B. terrestris*	5.98	1.107	5.40	< 0.001
		*A. vaga–C. cunicularius*	5.27	1.183	4.46	< 0.001
		*A. vaga–M. rotundata*	− 22.27	6.691	− 3.33	0.011
		*B. terrestris–M. rotundata*	− 28.25	6.633	− 4.26	< 0.001
		*B. terrestris–O. bicornis*	− 5.28	0.487	− 10.84	< 0.001
		*B. terrestris–O. cornuta*	− 3.87	0.653	− 5.93	< 0.001
		*C. cunicularius–M. rotundata*	− 27.54	6.657	− 4.14	< 0.001
		*C. cunicularius–O. bicornis*	− 4.57	0.780	− 5.87	< 0.001
		*C. cunicularius–O. cornuta*	− 3.16	0.922	− 3.43	0.008
		*M. rotundata–O. bicornis*	22.97	6.608	3.48	0.007
		*M. rotundata–O. cornuta*	24.38	6.634	3.68	0.003
	48	*A. mellifera–A. vaga*	− 4.04	1.162	− 3.47	0.006
		*A. mellifera–O. bicornis*	− 4.48	0.765	− 5.86	< 0.001
		*A. mellifera–O. cornuta*	− 3.48	0.963	− 3.62	0.004
		*A. vaga–B. terrestris*	3.78	1.057	3.58	0.004
		*A. vaga–C. cunicularius*	5.45	1.335	4.08	< 0.001
		*B. terrestris–O. bicornis*	− 4.22	0.575	− 7.35	< 0.001
		*B. terrestris–O. cornuta*	− 3.23	0.800	− 4.03	< 0.001
		*C. cunicularius–O. bicornis*	− 5.90	1.133	− 5.21	< 0.001
		*C. cunicularius–O. cornuta*	− 4.90	1.304	− 3.76	0.002
	96	*A. mellifera–B. terrestris*	− 6.37	1.650	− 3.86	0.002
		*A. mellifera–O. bicornis*	− 8.46	1.590	− 5.33	< 0.001
		*A. mellifera–O. cornuta*	− 8.31	1.940	− 4.28	< 0.001
		*B. terrestris–C. cunicularius*	6.44	2.260	2.85	0.044
		*C. cunicularius–O. bicornis*	− 8.54	2.250	− 3.79	0.002
Moribund	2 (co)	*B. terrestris–M. rotundata*	− 2.85	0.920	− 3.10	0.028
	2 (zi)	*A. mellifera–O. bicornis*	2.61	0.874	2.99	0.032
		*B. terrestris–O. bicornis*	1.79	0.602	2.98	0.033
	24	*A. mellifera–M. rotundata*	− 3.07	1.001	− 3.07	0.031
		*A. mellifera–O. bicornis*	− 2.24	0.732	− 3.07	0.031
		*B. terrestris–M. rotundata*	− 3.14	0.824	− 3.81	0.002
		*B. terrestris–O. bicornis*	− 2.31	0.444	− 5.20	< 0.001
	48	*A. mellifera–O. bicornis*	− 3.15	0.792	− 3.97	< 0.001
		*A. vaga–B. terrestris*	2.43	0.766	3.18	0.016
		*B. terrestris–O. bicornis*	− 3.05	0.512	− 5.96	< 0.001
		*O. bicornis–O. cornuta*	1.49	0.512	2.92	0.035
	96	*A. mellifera–O. bicornis*	− 4.95	1.671	− 2.96	0.031
		*A. vaga–B. terrestris*	4.40	1.407	3.12	0.019
		*B. terrestris–O. bicornis*	− 4.53	1.065	− 4.26	< 0.001
		*O. bicornis–O. cornuta*	2.70	0.954	2.83	0.045
Cramps	2	*A. mellifera–A. vaga*	3.58	0.867	4.13	< 0.001
		*A. mellifera–B. terrestris*	6.08	0.647	9.40	< 0.001
		*A. mellifera–C. cunicularius*	6.23	0.878	7.09	< 0.001
		*A. mellifera–M. rotundata*	4.97	0.803	6.19	< 0.001
		*A. mellifera–O. bicornis*	1.95	0.602	3.22	0.020
		*A. mellifera–O. cornuta*	3.39	0.764	4.44	< 0.001
		*A. vaga–B. terrestris*	2.51	0.772	3.24	0.019
		*A. vaga–C. cunicularius*	2.65	0.827	3.20	0.021
		*B. terrestris–O. bicornis*	− 4.14	0.489	− 8.48	< 0.001
		*B. terrestris–O. cornuta*	− 2.69	0.643	− 4.18	< 0.001
		*C. cunicularius–O. bicornis*	− 4.29	0.783	− 5.48	< 0.001
		*C. cunicularius–O. cornuta*	− 2.84	0.934	− 3.03	0.036
		*M. rotundata–O. bicornis*	− 3.04	0.687	− 4.42	< 0.001
	24	*A. mellifera–O. bicornis*	− 2.92	0.531	− 5.50	< 0.001
		*A. vaga–B. terrestris*	2.81	0.671	4.19	< 0.001
		*A. vaga–C. cunicularius*	2.65	0.713	3.71	0.004
		*B. terrestris–M. rotundata*	− 3.57	0.835	− 4.28	< 0.001
		*B. terrestris–O. bicornis*	− 3.98	0.420	− 9.49	< 0.001
		*B. terrestris–O. cornuta*	− 2.74	0.564	− 4.85	< 0.001
		*C. cunicularius–M. rotundata*	− 3.40	1.021	− 3.34	0.014
		*C. cunicularius–O. bicornis*	− 3.81	0.684	− 5.57	< 0.001
		*C. cunicularius–O. cornuta*	− 2.57	0.862	− 2.98	0.041
	48	*A. mellifera–A. vaga*	− 7.57	1.157	− 6.54	< 0.001
		*A. mellifera–B. terrestris*	− 4.42	1.074	− 4.12	< 0.001
		*A. mellifera–C. cunicularius*	− 4.93	1.252	− 3.94	0.001
		*A. mellifera–O. bicornis*	− 8.22	1.000	− 8.22	< 0.001
		*A. mellifera–O. cornuta*	− 7.21	1.124	− 6.42	< 0.001
		*A. vaga–B. terrestris*	3.15	0.839	3.75	0.002
		*A. vaga–C. cunicularius*	2.64	0.884	2.99	0.029
		*B. terrestris–O. bicornis*	− 3.80	0.537	− 7.08	< 0.001
		*B. terrestris–O. cornuta*	− 2.79	0.704	− 3.96	< 0.001
		*C. cunicularius–O. bicornis*	− 3.29	0.849	− 3.88	0.001
	96	*A. mellifera–A. vaga*	− 19.20	2.490	− 7.72	< 0.001
		*A. mellifera–B. terrestris*	− 15.38	2.410	− 6.39	< 0.001
		*A. mellifera–C. cunicularius*	− 16.57	2.690	− 6.17	< 0.001
		*A. mellifera–O. bicornis*	− 18.82	2.230	− 8.46	< 0.001
		*A. mellifera–O. cornuta*	− 18.27	2.430	− 7.52	< 0.001
		*B. terrestris–O. bicornis*	− 3.44	1.040	− 3.31	0.010
Affected	2	*A. mellifera–C. cunicularius*	− 3.24	0.632	− 5.13	< 0.001
		*A. mellifera–O. bicornis*	3.48	0.720	4.83	< 0.001
		*A. vaga–C. cunicularius*	− 7.95	1.875	− 4.24	< 0.001
		*A. vaga–M. rotundata*	− 5.75	1.934	− 2.97	0.040
		*B. terrestris–C. cunicularius*	− 3.11	0.608	− 5.12	< 0.001
		*B. terrestris–O. bicornis*	3.60	0.696	5.17	< 0.001
		*C. cunicularius–O. bicornis*	6.72	0.840	8.00	< 0.001
		*C. cunicularius–O. cornuta*	4.51	0.843	5.35	< 0.001
		*M. rotundata–O. bicornis*	4.52	0.873	5.18	< 0.001
	24	*A. mellifera–O. bicornis*	1.99	0.506	3.92	0.002
		*B. terrestris–O. bicornis*	1.42	0.465	3.06	0.030
	48	*A. mellifera–C. cunicularius*	8.09	2.186	3.70	0.002
		*A. vaga–C. cunicularius*	7.45	2.310	3.22	0.013
		*B. terrestris–C. cunicularius*	6.77	2.173	3.12	0.018
		*B. terrestris–O. cornuta*	− 2.07	0.619	− 3.34	0.009
		*C. cunicularius–O. bicornis*	− 7.73	2.183	− 3.54	0.004
		*C. cunicularius–O. cornuta*	− 8.84	2.220	− 3.98	< 0.001
	96	*A. mellifera–C. cunicularius*	19.92	4.507	4.42	< 0.001
		*A. vaga–B. terrestris*	6.42	2.164	2.97	0.028
		*A. vaga–C. cunicularius*	23.51	4.905	4.79	< 0.001
		*B. terrestris–C. cunicularius*	17.09	4.483	3.81	0.002
		*B. terrestris–O. bicornis*	− 5.72	1.186	− 4.83	< 0.001
		*B. terrestris–O. cornuta*	− 5.68	1.222	− 4.65	< 0.001
		*C. cunicularius–O. bicornis*	− 22.81	4.571	− 4.99	< 0.001
		*C. cunicularius–O. cornuta*	− 22.77	4.572	− 4.98	< 0.001

Estimates of binomial mixed effects model contrasts, their standard errors (SE) and z-ratios are only displayed for the *species* (seven levels) comparisons, which were significant at a level of alpha = 0.05. The post hoc analysis used multivariate t distribution adjustment (mvt) to account for multiple testing. Level “*M. rotundata*” was not included in analyses at the 96 h sampling time, because all individuals were dead 48 h after application. Results are given on the log odds ratio scale, which was used in the tests.

*co* conditional part of zero-inflated model, *zi* zero-inflated part of zero-inflated model.

**Table 4:** Mean proportion of alive bee individuals (± standard error) per tested species, which showed abnormal behavior (classified as four categories, where “symptoms” included the other three categories) in the control and insecticide treatment 2, 4, 24, 48, 72 and 96 h after application.

Behavior	Species	2 h	4 h	24 h	48 h	72 h	96 h
Control							
Symptoms	*A. mellifera*	0 (0)	0 (0)	0 (0)	0 (0)	0 (0)	0 (0)
	*A. vaga*	0 (0)	0 (0)	0 (0)	0.02 (0.022)	0 (0)	0 (0)
	*B. terrestris*	0.01 (0.006)	0.01 (0.006)	0 (0)	0 (0)	0 (0)	0 (0)
	*C. cunicularius*	0 (0)	0.02 (0.022)	0 (0)	0 (0)	0 (0)	0 (0)
	*M. rotundata*	0 (0)	0 (0)	0 (0)	0 (0)	0 (0)	0 (0)
	*O. bicornis*	0.01 (0.006)	0 (0)	0.02 (0.015)	0.01 (0.008)	0 (0)	0.01 (0.01)
	*O. cornuta*	0 (0)	0 (0)	0.02 (0.017)	0 (0)	0 (0)	0.03 (0.022)
Moribund	*A. mellifera*	0 (0)	0 (0)	0 (0)	0 (0)	0 (0)	0 (0)
	*A. vaga*	0 (0)	0 (0)	0 (0)	0 (0)	0 (0)	0 (0)
	*B. terrestris*	0 (0)	0.01 (0.006)	0 (0)	0 (0)	0 (0)	0 (0)
	*C. cunicularius*	0 (0)	0.02 (0.022)	0 (0)	0 (0)	0 (0)	0 (0)
	*M. rotundata*	0 (0)	0 (0)	0 (0)	0 (0)	0 (0)	0 (0)
	*O. bicornis*	0.01 (0.006)	0 (0)	0 (0)	0.01 (0.008)	0 (0)	0 (0)
	*O. cornuta*	0 (0)	0 (0)	0.02 (0.017)	0 (0)	0 (0)	0 (0)
Cramps	*A. mellifera*	0 (0)	0 (0)	0 (0)	0 (0)	0 (0)	0 (0)
	*A. vaga*	0 (0)	0 (0)	0 (0)	0.02 (0.022)	0 (0)	0 (0)
	*B. terrestris*	0.01 (0.006)	0 (0)	0 (0)	0 (0)	0 (0)	0 (0)
	*C. cunicularius*	0 (0)	0 (0)	0 (0)	0 (0)	0 (0)	0 (0)
	*M. rotundata*	0 (0)	0 (0)	0 (0)	0 (0)	0 (0)	0 (0)
	*O. bicornis*	0 (0)	0 (0)	0 (0)	0 (0)	0 (0)	0.01 (0.008)
	*O. cornuta*	0 (0)	0 (0)	0 (0)	0 (0)	0 (0)	0 (0)
Affected	*A. mellifera*	0 (0)	0 (0)	0 (0)	0 (0)	0 (0)	0 (0)
	*A. vaga*	0 (0)	0 (0)	0 (0)	0 (0)	0 (0)	0 (0)
	*B. terrestris*	0 (0)	0 (0)	0 (0)	0 (0)	0 (0)	0 (0)
	*C. cunicularius*	0 (0)	0 (0)	0 (0)	0 (0)	0 (0)	0 (0)
	*M. rotundata*	0 (0)	0 (0)	0 (0)	0 (0)	0 (0)	0 (0)
	*O. bicornis*	0 (0)	0 (0)	0.02 (0.015)	0 (0)	0 (0)	0.01 (0.006)
	*O. cornuta*	0 (0)	0 (0)	0 (0)	0 (0)	0 (0)	0.03 (0.022)
Insecticide treatment							
Symptoms	*A. mellifera*	0.94 (0.027)	0.99 (0.007)	0.63 (0.091)	0.05 (0.035)	0 (0)	0 (0)
	*A. vaga*	1 (0)	1 (0)	1 (0)	0.50 (0.164)	0.33 (0.211)	0.20 (0.200)
	*B. terrestris*	0.27 (0.062)	0.35 (0.064)	0.27 (0.054)	0.11 (0.042)	0.04 (0.023)	0 (0)
	*C. cunicularius*	0.92 (0.057)	0.83 (0.063)	0.21 (0.094)	0 (0)	0.13 (0.111)	0 (0)
	*M. rotundata*	0.51 (0.146)	0.77 (0.084)	1 (0)	NA	NA	NA
	*O. bicornis*	0.98 (0.013)	0.96 (0.027)	0.85 (0.052)	0.58 (0.070)	0.25 (0.059)	0.15 (0.046)
	*O. cornuta*	0.88 (0.067)	0.87 (0.071)	0.62 (0.126)	0.41 (0.131)	0.20 (0.107)	0.17 (0.094)
Moribund	*A. mellifera*	0.02 (0.016)	0.02 (0.017)	0.03 (0.018)	0.05 (0.035)	0 (0)	0 (0)
	*A. vaga*	0.36 (0.109)	0.27 (0.100)	0.15 (0.063)	0.31 (0.162)	0 (0)	0.20 (0.200)
	*B. terrestris*	0.02 (0.010)	0.04 (0.022)	0.09 (0.035)	0.03 (0.016)	0.01 (0.010)	0 (0)
	*C. cunicularius*	0.24 (0.128)	0.23 (0.091)	0 (0)	0 (0)	0.02 (0.022)	0 (0)
	*M. rotundata*	0.23 (0.121)	0.39 (0.120)	0.48 (0.190)	NA	NA	NA
	*O. bicornis*	0.19 (0.056)	0.25 (0.052)	0.21 (0.056)	0.11 (0.035)	0.11 (0.039)	0.10 (0.039)
	*O. cornuta*	0.20 (0.089)	0.14 (0.046)	0.38 (0.094)	0.07 (0.037)	0.05 (0.033)	0.04 (0.042)
Cramps	*A. mellifera*	0.92 (0.030)	0.98 (0.018)	0.27 (0.099)	0 (0)	0 (0)	0 (0)
	*A. vaga*	0.64 (0.109)	0.73 (0.100)	0.85 (0.063)	0.06 (0.063)	0.33 (0.211)	0 (0)
	*B. terrestris*	0.16 (0.055)	0.24 (0.051)	0.09 (0.037)	0.02 (0.013)	0 (0)	0 (0)
	*C. cunicularius*	0.36 (0.132)	0.25 (0.093)	0.18 (0.076)	0 (0)	0.11 (0.111)	0 (0)
	*M. rotundata*	0.10 (0.043)	0.38 (0.091)	0.31 (0.156)	NA	NA	NA
	*O. bicornis*	0.79 (0.056)	0.71 (0.059)	0.62 (0.079)	0.41 (0.068)	0.07 (0.037)	0.05 (0.028)
	*O. cornuta*	0.63 (0.112)	0.68 (0.090)	0.08 (0.032)	0.21 (0.077)	0.13 (0.090)	0.02 (0.021)
Affected	*A. mellifera*	0 (0)	0 (0)	0.45 (0.098)	0 (0)	0 (0)	0 (0)
	*A. vaga*	0 (0)	0 (0)	0 (0)	0.13 (0.082)	0 (0)	0 (0)
	*B. terrestris*	0.08 (0.030)	0.07 (0.023)	0.09 (0.038)	0.06 (0.030)	0.03 (0.021)	0 (0)
	*C. cunicularius*	0.32 (0.143)	0.35 (0.101)	0.03 (0.028)	0 (0)	0 (0)	0 (0)
	*M. rotundata*	0.22 (0.119)	0 (0)	0.31 (0.156)	NA	NA	NA
	*O. bicornis*	0.01 (0.006)	0 (0)	0.04 (0.020)	0.13 (0.046)	0.07 (0.027)	0 (0)
	*O. cornuta*	0.05 (0.026)	0.05 (0.026)	0.16 (0.053)	0.32 (0.123)	0.03 (0.028)	0.10 (0.084)

We do not report any behavioral data for *M. rotundata* in the insecticide treatment at 48, 72 and 96 h, because all individuals were dead 48 h after application.

Abnormal behavior of *M. rotundata* was difficult to evaluate due to the small number of individuals that survived the first 24 h (Supplement 2); behavior could not be measured after 24 h until the end of the experiment, because all bee individuals had already died at this point. While bees experienced moribund behavior and cramps right after insecticide application within 24 h, we observed a peak in restless and uncoordinated behavior, vertigo, and bees at a dorsal position (i.e., “affected” behavior) later between 24 and 48 h after application (Fig. 4, Supplement 12) except for *C. cunicularius*, which revealed 32% (35%) individuals with affected behavior two (four) hours after application (Fig. 4, Table 4).

## Discussion

Our findings revealed that honey bees respond more sensitively to a lambda-cyhalothrin exposure than some, but not all of the other bee species tested in our trials. Particularly individuals of the second social species, *B. terrestris*, as well as individuals of one of the mason bee species, *O. bicornis,* showed a significantly lower mortality than the honey bee. For these and the three other species, whose mortality was not significantly different from the honey bee, honey bee data could be a direct substitute for investigating sensitivity to lambda-cyhalothrin in bees. This would not take into account the smallest species in our experiment, *M. rotundata,* which displayed higher mortality rates than *A. mellifera.* Nor would it factor in the significant differences in sublethal effects between honey bees and the non-*Apis* species we investigated.

Species in our study showed specific behavioral profiles after they were treated with lambda-cyhalothrin. For example, *A. mellifera* was primarily cramping right after application, *C. cunicularius* mainly restless, uncoordinated, with vertigo or in dorsal position (i.e., being affected), *M. rotundata* showed moribund individuals that eventually all died, and *O. bicornis* was mainly cramping or displayed a moribund behavior until the end of the experimental period. Since such behavioral differences are qualitative, they are difficult to compare to each other. The status moribund may be more closely related to lethality than the other categories^50^, yet even moribund individuals may recover. Complementary to the lethal effects, *B. terrestris* individuals showed sublethal effects after insecticide treatment, but significantly less often than most other species in our study. Even when only a small percentage of bees experience sublethal effects after lambda-cyhalothrin exposure, it may still be potentially relevant in the field; bumble bees that were exposed to the insecticide in a semi-field study showed changes in pollen foraging behavior^51^, an indication for sublethal effects.

Behavioral abnormalities in our study did not last longer than 24–48 h after application in half of the species, including the honey bee and bumble bee species. Though, sublethal effects in *O. bicornis* (and for single behavioral categories also *O. cornuta* and *A. vaga*) were observed over a longer period. While bees in the laboratory may eventually recover, abnormal behavior in a field scenario may potentially lead to mortality due to predation or other stressors. In addition, individuals may be exposed repeatedly, which amplifies effects. Indeed, behavioral changes e.g. in *O. bicornis* were found to be also related to the number of lambda-cyhalothrin exposure events under semi-field conditions^52^. Our results highlight that considering mortality on its own may bear the risk of underestimating the full extent of sensitivity. Lethal and sublethal effects are complementary aspects^53,54^. Hence, it is surprising that sublethal effects in bees have been investigated for less than one third of pesticides^54^. The majority of these data covers honey bees^54,55^.

Differential sensitivity to pesticide exposure across bee species depends on both variability in specific detoxification capacities and differences in body size^19,24^. In our study, we did not measure detoxification capacities but average bee weight. Size/weight of individual bees within the same bee species is postulated to negatively correlate with mortality rates after pesticide exposure in laboratory experiments^18,56,57^ (but cf. reference^58^), since a larger body mass potentially buffers a defined volume of pesticide more easily than a smaller mass. Larger bee individuals also have a smaller body surface area to body volume ratio and therefore experience lower levels of contact exposure^19^. Our results support these postulations. For the bee species tested in our experiment, average bee weight was positively, non-linearly related to average bee survival in the insecticide treatment but not in the control (see Supplements 8 and 9). The heaviest species *B. terrestris* (mean: 253 mg) was the least susceptible whereas the lightest species *M. rotundata* (mean: 40 mg) the most susceptible to lambda-cyhalothrin exposure; other species weights (with one exception) are also in line with this hypothesis (cf. Supplement 8). While bee weight or size may be used as one proxy for bee sensitivity, mortality rates and sublethal effects of pesticide-exposed bees are insufficiently explained by individual weight alone^58–61^. An alternative approach to explain differences in bee sensitivity is to link it with phylogenetic and toxicogenomic information^62^, which reflects specific detoxification capacities^63–66^. Genes from the P450 complex play an important role in the detoxification of xenobiotics like pyretroids^67–69^. Although we did not investigate gene activity, it might explain in part, why *M. rotundata* (which lacks P450 enzymes^70^) showed high mortality in our experiment.

Differential sensitivity of a bee species does not fully describe its vulnerability, which also encompasses exposure and recovery from exposure^71,72^. While our experiment focused on bee sensitivity in response to topical lambda-cyhalothrin at a field-realistic rate, our tested bee species likely experience different exposure routes to the insecticide in the field due to their different life histories, foraging behavior, and sociality (Fig. 1). Life history traits may modulate exposure probabilities in a complex way^73^. Some of the species that we tested in the laboratory may be less exposed to lambda-cyhalothrin in the field, since they use specific non-crop food sources (e.g., *Salix* spec. in the case of *A. vaga*^74^). Nevertheless, these species may still nest in vicinity to agricultural crops, which may result in an exposure risk for one individual nest or a whole population (in the case of aggregations^75^ of e.g., *A. vaga* or *C. cunicularius*). Pesticides (e.g., also those with lambda-cyhalothrin) can be sprayed throughout the season and may drift to these adjacent areas repeatedly. Bee species like honey bees that prefer mass-flowering crops may even be sprayed while foraging on these crops. We did not address exposure routes in our study but semi-field experiments with two of our tested species have shown that these aspects (e.g., multiple spraying) can modulate bee vulnerability to lambda-cyhalothrin (reducing pollen foraging^51^, inducing an excitatory effect^52^). The availability of life history trait data of wild bee species is still too limited to use them as predictors in analysis; potentially important traits (such as e.g., grooming behavior^76^ or hairiness^77^) may not have been studied yet, or traits are solely estimated via congeneric representatives rather than being empirically measured^78–80^. Hence, species-specific toxicity profiles are complex and difficult to compile. In addition, bee individuals of the same species may display different sensitivities to pesticides in response to their sex^81^ (but see reference^61^) or developmental stage and age^82,83^. While we only included young adult female bees, age standardization was challenging, particularly for bees caught in the wild, so that we cannot rule out that senescence may have had an impact on our results.

In the two social bee species we tested, exposure of individual worker bees in the field may be compensated by the colony and its regulation mechanisms^84^, for example, through trophallaxis in honey bees^85^ or mixing of nectar/pollen (but see reference^73^). This may also decrease the probability of exposure of reproducing individuals (queens and males). Queens of social species may still encounter pesticides during certain life stages, e.g. hibernation^86^ or colony founding^87^, although we are not aware of any studies on lambda-cyhalothrin in this context. We did not test queens of either honey bees or bumble bees in our experiment. Comparative studies on bumble bee queen and worker mortalities with other pesticides revealed that queens were less sensitive than workers due to not only their greater size but probably also to their thicker cuticle and higher fat body reserves, which express P450 detoxification enzymes^61^. Sublethal effects and mortality of non-reproducing workers in our two social species may not directly correspond to the fitness of their colonies, as long as a sufficient number of nest mates are alive and not affected. In contrast, sensitivity of the solitary species used in our trials is likely linked directly to their reproductive success.

In our study we focussed on exposure via direct contact. This exposure route is one of the major sources of xenobiotic intake in bees^19,88^. Our application method mimicked field-realistic techniques and thereby showed increased mortality and sublethal effects after application of an insecticide containing lambda-cyhalothrin, which is approved for use in bee-attractive crops during flowering. However, this method simulates a worst case scenario in the laboratory, and it is usually unlikely that laboratory effects would translate into equivalent effects in the field^89^. Our application method mists the whole insect with pesticide spray, which makes it different from the standardized test method currently suggested by OECD guidelines (droplet application on the thorax^30,31^). Sensitivity results are therefore difficult to compare to other studies, which usually calculate LD_50_ values (median lethal dose, at which 50% of the test subjects die after a specified test duration). However, our approach enables us to describe the potential risk of contact exposure to bees and also indicates the relative sensitivity of the tested bee species to each other. Whether the patterns observed in our study can be reproduced using other pesticides needs to be confirmed in future studies. The lack of knowledge on a wider range of active substance classes and modes of action calls for more research in this area^90,91^. By including a wider range of species, we measured the differences in sensitivity more directly^57^ than meta-analysis on multiple studies of single species would do. In general, handling more than one bee species is time-consuming, and experimental set-up is challenging, particularly when using bees from natural populations. Bees caught from wild populations probably have experienced other environmental stressors (e.g., parasites, nutritional status) than commercially reared bees. This makes comparisons with and standardizations to managed bee species more complex or even impossible (e.g. when lifespans do not overlap). Nevertheless, inclusion of multiple species in the same experiment is necessary to identify additional model species for covering a wider range of exposure scenarios. This provides an avenue for a more generalized risk assessment, which includes a compilation of study species that resemble each other in some characteristics but vary in others.

## Conclusion

Our results on the sensitivity, i.e. mortality and behavioral abnormalities, of seven social and solitary bee species to the active substance lambda-cyhalothrin showed inter-specific differences in bee sensitivity. The risk of the pesticide to the tested non-*Apis* bees was partly explained by differences in weight but not other life history traits. If risk assessment based on *A. mellifera* is protective of a wider range of bee species is still a controversy (e.g., reference^92^ in response to reference^58^); our data supports the idea that honey bees can be surrogate for some bee species’ sensitivity. However, establishing a surrogacy in risk assessment depends on proposed safety factors, which often cannot be reliably set due to a lack of bee species-specific data (cf. reference^11^). Particularly data and analyses on sublethal effects are lacking for the majority of pesticides^54^. Even if we could estimate a safety factor based on our data of species’ responses at the individual level, responses may diverge at the reproductive level (i.e., when considering colonies versus individuals)^93^ or for different developmental stages or casts^61^. Hence, for adequately covering more sensitive species and a wider range of vulnerability*,* a larger set of species may be useful to consider for risk assessment. This may require further testing refinements in cases where species are not commercially available.

To realistically estimate vulnerability of existing bee communities in a field setting by using model organisms, further knowledge and data on key life history traits as well as sensitivity are needed. Our study showed that multi-species experiments are challenging but possible and should be promoted whenever possible, even if certain obstacles (reliable sources, standardization of age) are difficult to circumvent. Test methods should be adjusted and optimized for other wild bee species at laboratory level in order to establish them as additional or even standard models in the risk assessment besides the honey bee. In addition, data and statistical analyses of behavioral abnormalities should become a requirement rather than an option in studies on bee sensitivity, particularly since the method of their assessment is already implemented in the current OECD guidelines. Their statistical evaluation provides valuable additional information on the sensitivity and hence vulnerability of bee species to pesticides^54^.

### Supplementary Information

Supplementary Information.

## Data Availability

The datasets supporting this study are available in the OpenAgrar repository (https://doi.org/10.5073/20231124-160110-0)^94^.
