# Supplementary material for: Risk assessment requires several bee species to address species-specific sensitivity to insecticides at field-realistic concentrations
Source: Sci Rep. 2023 Dec 18;13:22533. doi: 10.1038/s41598-023-48818-7 (PMC10728145; doi:10.1038/s41598-023-48818-7)
Supplement: Supplementary file 1 — Supplementary Information. [file 41598_2023_48818_MOESM1_ESM.docx]

# Supplements to “Risk assessment requires several bee species to address species-specific sensitivity to insecticides at field-realistic concentrations”

Tobias Jütte, Anna Wernecke, Felix Klaus, Jens Pistorius and Anke C. Dietzsch

**Supplement 1.** Location and geographical coordinates where bee individuals of *Andrena vaga* and *Colletes cunicularius* were caught in the wild in April 2018 (trial 1) and April 2019 (trial 5).

| Bee species | Trial no. | Location | Coordinates |
| --- | --- | --- | --- |
| *Andrena vaga* | 1 | Messeweg, 38104 Braunschweig, Germany | 52°16'36.04"N  10°34'7.01"E |
|  | 5 | Messeweg, 38104 Braunschweig, Germany | 52°16'36.04"N  10°34'7.01"E |
|  |  | Schillstr., 38102 Braunschweig, Germany | 52°15'23.34"N  10°32'28.49"E |
|  |  | Glogaustr. 12, 38124 Braunschweig, Germany | 52°13'35.34"N  10°31'21.23"E |
|  |  | Theisenstr. 49-51, 38108 Braunschweig, Germany | 52°17'44.68"N  10°32'7.04"E |
| *Colletes cunicularius* | 1 | Messeweg, 38104 Braunschweig, Germany | 52°16'35.51"N  10°34'0.48"E |
|  | 5 | Messeweg, 38104 Braunschweig, Germany | 52°16'35.51"N  10°34'0.48"E |
|  |  | Glogaustr. 12, 38124 Braunschweig, Germany | 52°13'36.25"N  10°31'26.16"E |
|  |  | Vossenkamp, 38104 Braunschweig, Germany | 52°17'9.22"N  10°32'59.72"E |
|  |  | Schrotweg, 38122 Braunschweig, Germany | 52°14'21.98"N  10°30'58.61"E |
|  |  | Theisenstr. 49-51, 38108 Braunschweig, Germany | 52°17'44.68"N  10°32'7.04"E |

**Supplement 2.** Mean cumulative mortality over the course of six trials with seven bee species. Cages with bees were assigned to one of three treatments: water (control; C), Karate® Zeon (lambda-cyhalothrin; T) or Dandadim^©^ Progress (dimethoate; TOX). Number of dead bees were counted after 2, 4, 24, 48, 72 and 96 hours, respectively. N_C_=number of cages, N_B_=number of bees within cages of a particular treatment, bee species and trial, Weight ±SE= average weight of one bee measured on a cage level ±standard error in mg, NA=not available; for A. mellifera we assumed a weight of 100 mg per bee as cited in the literature^1^.

| Trial no. (Time) | Bee  species | Treat-ment | N_C_ | N_B_ | Weight ±SE [mg] | Mean cumulative mortality [%] | | | | | |
| --- | --- | --- | --- | --- | --- | --- | --- | --- | --- | --- | --- |
|  |  |  |  |  |  | +2 h | +4 h | +24 h | +48 h | +72 h | +96 h |
| 1 (Apr 2018) | *Apis mellifera* | C | 2 | 20 | NA | 0.0 | 0.0 | 0.0 | 0.0 | 0.0 | 0.0 |
|  |  | T | 3 | 29 | NA | 3.3 | 16.7 | 48.1 | 58.1 | 68.5 | 68.5 |
|  |  | TOX | 1 | 10 | NA | 0.0 | 90.0 | 100.0 | 100.0 | 100.0 | 100.0 |
|  | *Bombus terrestris* | C | 3 | 15 | 240±3.0 | 0.0 | 0.0 | 0.0 | 0.0 | 0.0 | 6.7 |
|  |  | T | 3 | 15 | 228±5.2 | 0.0 | 0.0 | 13.3 | 26.7 | 30.0 | 33.3 |
|  |  | TOX | 1 | 5 | 210 | 0.0 | 0.0 | 100.0 | 100.0 | 100.0 | 100.0 |
|  | *Osmia bicornis* | C | 3 | 15 | 160±1.0 | 0.0 | 0.0 | 0.0 | 0.0 | 0.0 | 0.0 |
|  |  | T | 3 | 15 | 160±0.7 | 0.0 | 0.0 | 0.0 | 0.0 | 6.7 | 6.7 |
|  |  | TOX | 1 | 5 | 159 | 0.0 | 0.0 | 80.0 | 100.0 | 100.0 | 100.0 |
|  | *Colletes cunicularius* | C | 3 | 15 | 136±9.7 | 0.0 | 0.0 | 6.7 | 6.7 | 6.7 | 13.3 |
|  |  | T | 3 | 13 | 139±4.5 | 0.0 | 0.0 | 15.0 | 21.7 | 30.0 | 30.0 |
|  |  | TOX | 1 | 5 | 143 | 0.0 | 40.0 | 80.0 | 100.0 | 100.0 | 100.0 |
|  | *Andrena vaga* | C | 3 | 15 | 130±4.7 | 0.0 | 0.0 | 0.0 | 0.0 | 0.0 | 0.0 |
|  |  | T | 3 | 15 | 133±4.1 | 0.0 | 13.3 | 13.3 | 53.3 | 53.3 | 53.3 |
|  |  | TOX | 1 | 5 | 126 | 0.0 | 80.0 | 100.0 | 100.0 | 100.0 | 100.0 |
| 2 (Jun 2018) | *Apis mellifera* | C | 3 | 30 | NA | 0.0 | 0.0 | 0.0 | 0.0 | 0.0 | 0.0 |
|  |  | T | 3 | 30 | NA | 0.0 | 0.0 | 13.3 | 13.3 | 13.3 | 13.3 |
|  |  | TOX | 1 | 10 | NA | 0.0 | 60.0 | 100.0 | 100.0 | 100.0 | 100.0 |
|  | *Bombus terrestris* | C | 6 | 30 | 281±5.0 | 0.0 | 0.0 | 0.0 | 0.0 | 0.0 | 0.0 |
|  |  | T | 6 | 30 | 278±4.5 | 0.0 | 0.0 | 23.3 | 26.7 | 26.7 | 26.7 |
|  |  | TOX | 2 | 10 | 283±3.5 | 0.0 | 0.0 | 70.0 | 90.0 | 90.0 | 90.0 |
|  | *Osmia bicornis* | C | 6 | 30 | 142±1.2 | 0.0 | 0.0 | 0.0 | 0.0 | 0.0 | 0.0 |
|  |  | T | 6 | 30 | 143±1.5 | 0.0 | 0.0 | 0.0 | 0.0 | 0.0 | 13.6 |
|  |  | TOX | 2 | 10 | 146±1.2 | 0.0 | 0.0 | 100.0 | 100.0 | 100.0 | 100.0 |
|  | *Osmia cornuta* | C | 6 | 30 | 140±0.6 | 0.0 | 0.0 | 0.0 | 0.0 | 3.3 | 3.3 |
|  |  | T | 6 | 29 | 136±1.3 | 0.0 | 0.0 | 12.8 | 26.1 | 39.4 | 72.5 |
|  |  | TOX | 2 | 10 | 142±0.8 | 0.0 | 0.0 | 100.0 | 100.0 | 100.0 | 100.0 |
| 3 (Jul 2018) | *Apis mellifera* | C | 3 | 30 | NA | 0.0 | 0.0 | 0.0 | 0.0 | 0.0 | 3.3 |
|  |  | T | 3 | 30 | NA | 13.3 | 20.0 | 63.3 | 80.0 | 83.3 | 83.3 |
|  |  | TOX | 1 | 10 | NA | 40.0 | 100.0 | 100.0 | 100.0 | 100.0 | 100.0 |
|  | *Bombus terrestris* | C | 6 | 30 | 210±5.4 | 0.0 | 0.0 | 3.3 | 3.3 | 3.3 | 3.3 |
|  |  | T | 6 | 30 | 216±4.0 | 0.0 | 0.0 | 0.0 | 3.3 | 3.3 | 6.7 |
|  |  | TOX | 2 | 10 | 216±28.3 | 0.0 | 0.0 | 80.0 | 90.0 | 90.0 | 90.0 |
|  | *Osmia bicornis* | C | 6 | 28 | 149±0.8 | 0.0 | 0.0 | 0.0 | 0.0 | 0.0 | 0.0 |
|  |  | T | 6 | 30 | 149±0.8 | 0.0 | 0.0 | 0.0 | 0.0 | 0.0 | 0.0 |
|  |  | TOX | 2 | 10 | 147±0.6 | 0.0 | 0.0 | 90.0 | 100.0 | 100.0 | 100.0 |
|  | *Megachile rotundata* | C | 6 | 30 | 37±0.2 | 3.3 | 3.3 | 3.3 | 6.7 | 10.0 | 10.0 |
|  |  | T | 6 | 30 | 38±0.7 | 33.3 | 33.3 | 70.0 | 100.0 | 100.0 | 100.0 |
|  |  | TOX | 2 | 10 | 39±1.3 | 10.0 | 10.0 | 80.0 | 100.0 | 100.0 | 100.0 |
| 4 (Mar 2019) | *Apis mellifera* | C | 3 | 30 | NA | 0.0 | 0.0 | 0.0 | 0.0 | 0.0 | 0.0 |
|  |  | T | 3 | 30 | NA | 0.0 | 6.7 | 63.3 | 70.0 | 70.0 | 70.0 |
|  |  | TOX | 1 | 10 | NA | 0.0 | 50.0 | 100.0 | 100.0 | 100.0 | 100.0 |
|  | *Bombus terrestris* | C | 6 | 30 | 251±11.7 | 0.0 | 0.0 | 0.0 | 0.0 | 0.0 | 0.0 |
|  |  | T | 6 | 30 | 238±10.1 | 0.0 | 0.0 | 3.3 | 10.0 | 10.0 | 10.0 |
|  |  | TOX | 2 | 10 | 250±23.5 | 0.0 | 0.0 | 50.0 | 50.0 | 50.0 | 50.0 |
|  | *Osmia bicornis* | C | 6 | 30 | 143±0.4 | 0.0 | 0.0 | 0.0 | 0.0 | 0.0 | 0.0 |
|  |  | T | 6 | 30 | 143±0.7 | 0.0 | 0.0 | 0.0 | 3.3 | 6.7 | 20.0 |
|  |  | TOX | 2 | 10 | 142±0.2 | 0.0 | 0.0 | 100.0 | 100.0 | 100.0 | 100.0 |
|  | *Osmia cornuta* | C | 6 | 30 | 181±2.1 | 0.0 | 0.0 | 0.0 | 0.0 | 3.3 | 3.3 |
|  |  | T | 6 | 30 | 180±1.7 | 0.0 | 0.0 | 13.3 | 16.7 | 20.0 | 20.0 |
|  |  | TOX | 2 | 10 | 185±2.6 | 0.0 | 0.0 | 100.0 | 100.0 | 100.0 | 100.0 |
| 5 (Apr 2019) | *Apis mellifera* | C | 3 | 30 | NA | 0.0 | 0.0 | 0.0 | 0.0 | 3.3 | 10.0 |
|  |  | T | 3 | 30 | NA | 0.0 | 3.3 | 63.3 | 80.0 | 80.0 | 80.0 |
|  |  | TOX | 1 | 10 | NA | 20.0 | 70.0 | 100.0 | 100.0 | 100.0 | 100.0 |
|  | *Bombus terrestris* | C | 6 | 30 | 260±17.4 | 0.0 | 0.0 | 0.0 | 0.0 | 0.0 | 0.0 |
|  |  | T | 6 | 30 | 263±19.2 | 0.0 | 0.0 | 0.0 | 3.3 | 3.3 | 3.3 |
|  |  | TOX | 2 | 10 | 257±18.2 | 0.0 | 20.0 | 60.0 | 60.0 | 60.0 | 60.0 |
|  | *Osmia bicornis* | C | 6 | 30 | 144±0.5 | 0.0 | 0.0 | 6.7 | 6.7 | 6.7 | 10.0 |
|  |  | T | 6 | 30 | 144±0.6 | 0.0 | 0.0 | 6.7 | 30.0 | 40.0 | 50.0 |
|  |  | TOX | 2 | 10 | 144±0.5 | 0.0 | 0.0 | 100.0 | 100.0 | 100.0 | 100.0 |
|  | *Colletes cunicularius* | C | 6 | 30 | 125±3.4 | 0.0 | 0.0 | 6.7 | 6.7 | 6.7 | 10.0 |
|  |  | T | 6 | 30 | 124±3.5 | 0.0 | 0.0 | 20.0 | 33.3 | 33.3 | 40.0 |
|  |  | TOX | 2 | 10 | 136±13.0 | 0.0 | 0.0 | 90.0 | 100.0 | 100.0 | 100.0 |
|  | *Andrena vaga* | C | 6 | 30 | 121±1.8 | 0.0 | 0.0 | 6.7 | 6.7 | 10.0 | 16.7 |
|  |  | T | 6 | 30 | 121±3.0 | 0.0 | 0.0 | 40.0 | 73.3 | 83.3 | 90.0 |
|  |  | TOX | 2 | 10 | 129±4.7 | 0.0 | 0.0 | 50.0 | 100.0 | 100.0 | 100.0 |
| 6 (Jul 2019) | *Apis mellifera* | C | 3 | 30 | NA | 0.0 | 0.0 | 0.0 | 0.0 | 0.0 | 0.0 |
|  |  | T | 3 | 30 | NA | 0.0 | 0.0 | 33.3 | 43.3 | 43.3 | 46.7 |
|  |  | TOX | 1 | 10 | NA | 10.0 | 30.0 | 60.0 | 100.0 | 100.0 | 100.0 |
|  | *Bombus terrestris* | C | 6 | 30 | 279±8.2 | 0.0 | 0.0 | 0.0 | 0.0 | 3.3 | 3.3 |
|  |  | T | 6 | 30 | 277±7.7 | 0.0 | 0.0 | 0.0 | 0.0 | 0.0 | 0.0 |
|  |  | TOX | 2 | 10 | 287±12.9 | 0.0 | 0.0 | 0.0 | 0.0 | 0.0 | 0.0 |
|  | *Osmia bicornis* | C | 6 | 30 | 144±0.7 | 0.0 | 3.3 | 10.0 | 13.3 | 13.3 | 13.3 |
|  |  | T | 6 | 30 | 145±0.8 | 0.0 | 0.0 | 36.7 | 50.0 | 50.0 | 53.3 |
|  |  | TOX | 2 | 10 | 147±1.1 | 0.0 | 0.0 | 90.0 | 100.0 | 100.0 | 100.0 |
|  | *Megachile rotundata* | C | 6 | 30 | 42±0.4 | 0.0 | 0.0 | 3.3 | 3.3 | 3.3 | 6.7 |
|  |  | T | 6 | 30 | 42±0.5 | 0.0 | 0.0 | 83.3 | 100.0 | 100.0 | 100.0 |
|  |  | TOX | 2 | 10 | 42±0.4 | 0.0 | 0.0 | 80.0 | 100.0 | 100.0 | 100.0 |

**Supplement 3.** Methodological approach with species-specific modifications.

In the honey bees, no clinical symptoms of adult bee, bee larvae or any other brood diseases (e.g. *Nosema* and *Varroa* infestation) were visible during inspection before the trials. All other commercially obtained bees were visibly checked for symptoms at arrival. Before and during the trials, sugar solution was replaced daily, and filter paper lining of side walls, rear walls and cage floors (bumble bees without lining of side walls) were replaced when necessary. On the day of application, a pre-examination was performed to ensure that all bee individuals were vital and undamaged. In cases, experimental cages contained unfit bee individuals, they were replaced with extra cages containing only fit bees.

In trials with honey bees, 10 bee individuals were used in each of three replicate cages per treatment (i.e. 30 bees per treatment). In the trials with all other bee species, five bees were used in each of six replicate cages (i.e. 30 bees per treatment) in order to account for the species-specific lower density living conditions of these species. In 2018, only three replicate cages with each of five individuals were used for non-*Apis* species (i.e. 15 bee individuals per treatment). The weight of the different bee species, with exception of the honey bee (assuming an average weight of 100 mg per cage) and *Osmia* spp. (cocoons were weighed before assigning them to cages) was calculated as one weight measurement for all individuals in each cage. A few experimental cages included less bee individuals than described above (**cf. Supplement 2**) due to bee escapes during cage handling.

European honey bee *(Apis mellifera)*

Young adult worker honey bees were collected near the brood nest (estimated to be max. 10-12 days old, cf.^3^) from the brood chamber (normally from the 2^nd^ edge honey comb of the colony) one day before application, anesthetized with CO_2_, counted into standard test cages (10 bees per cage) and transferred to a climatic chamber overnight. Feeding was provided ad libitum with sugar solution (55 % in 2018, 50 % in 2019) via a 5-ml disposable syringe with a cut-off tip. Before application, honey bees were cooled down for approx. 2 h in a cooling chamber (4°C) until they were immobile, then transferred from the cages to petri dishes and directly sprayed in the spray chamber.

Buff-tailed bumble bee *(Bombus terrestris)*

Adult worker bumble bees of unknown age but similar sizes were removed from two (trial 1-5) or three (trial 6) colonies one day prior to application under red light using tweezers. They were transferred into standard test cages (5 bees per cage), which were placed into freezer bags and cooled down on crushed ice, until bees were immobile (approx. 4-10 min). Bee individuals were weighed per cage on an analytical laboratory scale and distributed equally to all treatments. Cages were then stored in a climatic chamber overnight. Feeding was provided ad libitum with sugar solution (55 % in 2018, 50 % in 2019) via a 10 ml disposable syringe with molten tip and perforations on the side. Immediately before application, bee individuals were cooled down per cage on crushed ice, transferred from the cages to petri dishes and directly sprayed in the spray chamber.

Red mason bee *(Osmia bicornis)* & European orchard bee *(Osmia cornuta)*

An incubation of three days for *Osmia cornuta* and seven days for *Osmia bicornis*, respectively, was carried out for hatching in order to identify vital individuals and to ensure a similar age of bee individuals (age: min. 1 days, max. 5 days old). Bee cocoons were cut open to determine sex as well as undeveloped, dead or parasitized individuals. Cocoons of female bees of the same weight class (using an analytical laboratory scale) were distributed equally to all treatments. Cocoons were fully cut open one day before application and bees were transferred to cages (5 bees per cage) in a climatic chamber overnight. Feeding was provided ad libitum with sugar solution (55 % in 2018, 50 % in 2019) via royal cups (400 µl). Immediately before application, bees were cooled down per cage at 4°C in a cooling chamber (approx. 2 h) in 2018 and in freezer bags on crushed ice (approx. 4-5 min) in 2019 until they were immobile. They were transferred to petri dishes and directly sprayed in the spray chamber.

Grey-backed mining bee *(Andrena vaga)* & Spring mining bee *(Colletes cunicularius)*

At the beginning of the activity period of wild populations, newly emerging individual females of these two species were caught (cf. Supplement 1) within ten days before the start of the trials and kept at 4°C. One day before application, they were weighed per cage with an analytical laboratory scale and distributed equally to all treatments. Bees were then transferred into a climatic chamber, with the temperature kept constant (2018) or the temperature gradually adjusted over several days (2019). Feeding was provided ad libitum with sugar solution (55 % in 2018, 50 % in 2019) via royal cups (400 µl). Immediately before application, bees were immobilized by cooling them either at 2°C in the refrigerator (approx. 2 h, 2018) or in freezer bags on crushed ice (approx. 4 min, 2019). They were transferred to petri dishes and directly sprayed in the spray chamber.

Alfalfa-leafcutting bee *(Megachile rotundata)*

Leaf-cutting bee cocoons were incubated at 30 °C and approximately 60 % r.F. in a climatic chamber in accordance with the recommendations of the Saskatchewan Alfalfa See Producers Association^4^. This procedure ensured that females used in the trials were vital and of a similar age (2018: median 6 days, min. 4 days, max. 8 days; 2019: median 8 days, min. 6 days, max. 11 days old) to each other and to the wild catches of the two mining bee species. Hatched bees were collected daily and stored at Ø 13°C until the start of the experiment. One day before application of the insecticide, females were cooled down in a climatic chamber at 4°C for approx. 1-2 minutes. Immobile bees were then weighed per cage with an analytical laboratory scale and equally distributed to all treatments. Cages were transferred to a climatic chamber overnight. Feeding was provided ad libitum with sugar solution (55 % in 2018, 50 % in 2019) via royal cups (400 µl). Immediately before application, bees were cooled per cage at 4°C for approx. 1-2 min, and immobile individuals were transferred to petri dishes and directly sprayed in the spray chamber.

**Supplement 4.** Model selection steps for the mixed effects Cox model. For comparison of the nested models and the stepwise reduction of the full Cox model, Log-Likelihood (logLik) and chi-square (Chi^2) statistics were used. Significant results at an alpha=0.05 level are shown in bold and indicate that the parameter should be included in the final model.

| **Models** | **logLik** | **Chi^2** | **Df** | **P** |
| --- | --- | --- | --- | --- |
| **Random component** |  |  |  |  |
| ~species * treatment + (1 \| trial #/cage) | -2066.9 |  |  |  |
| ~species * treatment + (1 \| trial #) | -2096.0 | 58.064 | 1 | **2.542e-14** |
|  |  |  |  |  |
| ~species * treatment + (1 \| trial #/cage) | -2066.9 |  |  |  |
| ~species * treatment + (1 \| cage) | -2067.5 | 1.1866 | 1 | 0.276 |
|  |  |  |  |  |
| ~species * treatment + (1 \| cage) | -2067.5 |  |  |  |
| ~species * treatment | -2102.7 | 70.304 | 1 | **< 2.2e-16** |
| **Fixed component** |  |  |  |  |
| ~species * treatment + (1 \| cage) | -2067.5 |  |  |  |
| ~species + treatment + (1 \| cage) | -2078.1 | 21.148 | 6 | **0.001726** |

- Factor *trial number* is not statistically significant for model but factor *cage* is
- Interaction between factors *species* and *treatment* is statistically significant for model
- Final model: mortality ~species * treatment + (1 | cage)

**Supplement 5.** Kaplan–Meier curves (survival over time) of bee individuals of different species treated with lambda-cyhalothrin (Karate® Zeon; black lines) and water (control; grey lines). Survival was significantly lower in the treatment compared to the control group for all species (based on mixed effects Cox models; cf. Table 1).

**
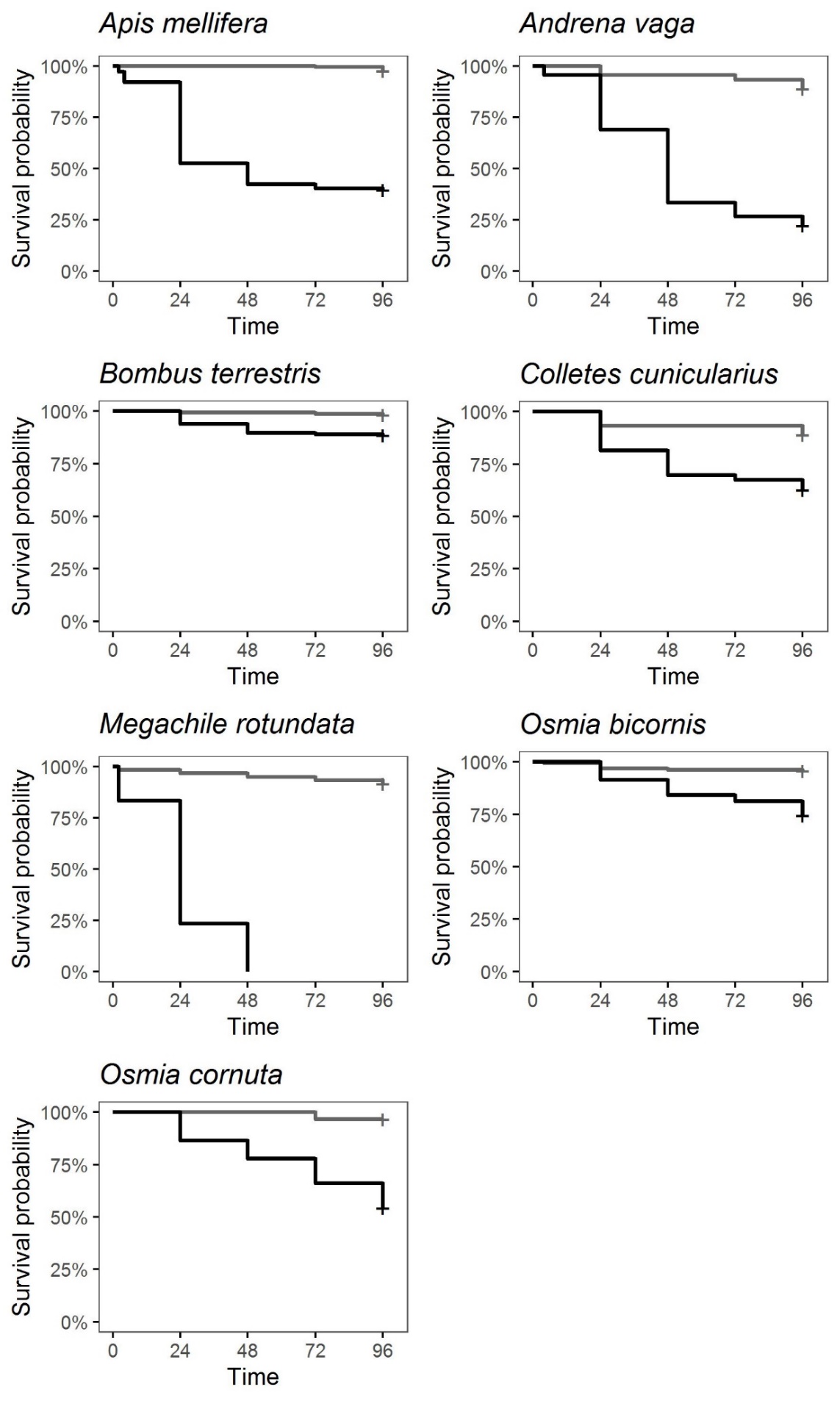
**

**Supplement 6.** Comparison of mortality between species for the control application. Estimates of mixed effects Cox model contrasts comparing control bees of all *species* (seven levels) with each other. The post hoc analysis used multivariate t distribution adjustment (mvt) to account for multiple testing. Results are given on the model scale (log scale).

| **Bee species contrasts** | **Estimate** | **Standard error** | **Z ratio** | **P value** |
| --- | --- | --- | --- | --- |
| *Apis mellifera - Andrena vaga* | -1.585 | 0.772 | -2.052 | 0.3763 |
| *Apis mellifera - Bombus terrestris* | 0.206 | 0.812 | 0.254 | 1.0000 |
| *Apis mellifera - Colletes cunicularius* | -1.653 | 0.767 | -2.154 | 0.3162 |
| *Apis mellifera - Megachile rotundata* | -1.302 | 0.757 | -1.719 | 0.5979 |
| *Apis mellifera - Osmia bicornis* | -0.662 | 0.685 | -0.966 | 0.9602 |
| *Apis mellifera - Osmia cornuta* | -0.391 | 0.932 | -0.419 | 0.9996 |
| *Bombus terrestris - Andrena vaga* | -1.791 | 0.808 | -2.218 | 0.2809 |
| *Bombus terrestris - Colletes cunicularius* | -1.859 | 0.803 | -2.316 | 0.2315 |
| *Bombus terrestris - Megachile rotundata* | -1.508 | 0.793 | -1.901 | 0.4738 |
| *Bombus terrestris - Osmia bicornis* | -0.868 | 0.725 | -1.198 | 0.8928 |
| *Bombus terrestris - Osmia cornuta* | -0.597 | 0.962 | -0.621 | 0.9960 |
| *Andrena vaga - Colletes cunicularius* | -0.068 | 0.763 | -0.089 | 1.0000 |
| *Andrena vaga - Megachile rotundata* | 0.283 | 0.753 | 0.376 | 0.9998 |
| *Andrena vaga - Osmia bicornis* | 0.923 | 0.680 | 1.357 | 0.8212 |
| *Andrena vaga - Osmia cornuta* | 1.194 | 0.929 | 1.286 | 0.8558 |
| *Colletes cunicularius - Megachile rotundata* | 0.351 | 0.748 | 0.470 | 0.9992 |
| *Colletes cunicularius - Osmia bicornis* | 0.991 | 0.675 | 1.469 | 0.7589 |
| *Colletes cunicularius - Osmia cornuta* | 1.262 | 0.924 | 1.365 | 0.8169 |
| *Megachile rotundata - Osmia bicornis* | 0.640 | 0.663 | 0.965 | 0.9604 |
| *Megachile rotundata - Osmia cornuta* | 0.911 | 0.916 | 0.994 | 0.9542 |
| *Osmia bicornis - Osmia cornuta* | 0.271 | 0.857 | 0.316 | 0.9999 |

**Supplements 7.** Model estimates of marginal mean hazard rates in the control group (a) and hazard rates in the treatment group (b) for all seven investigated bee species. Results are given on the respose scale (back-transformed from the log scale). Asymptotic confidence intervals (ACL, at a level of 95%) are shown as grey bars. Pair-wise comparisons are given in Supplement 6 (for a) and Table 2 (for b).

**(a)**


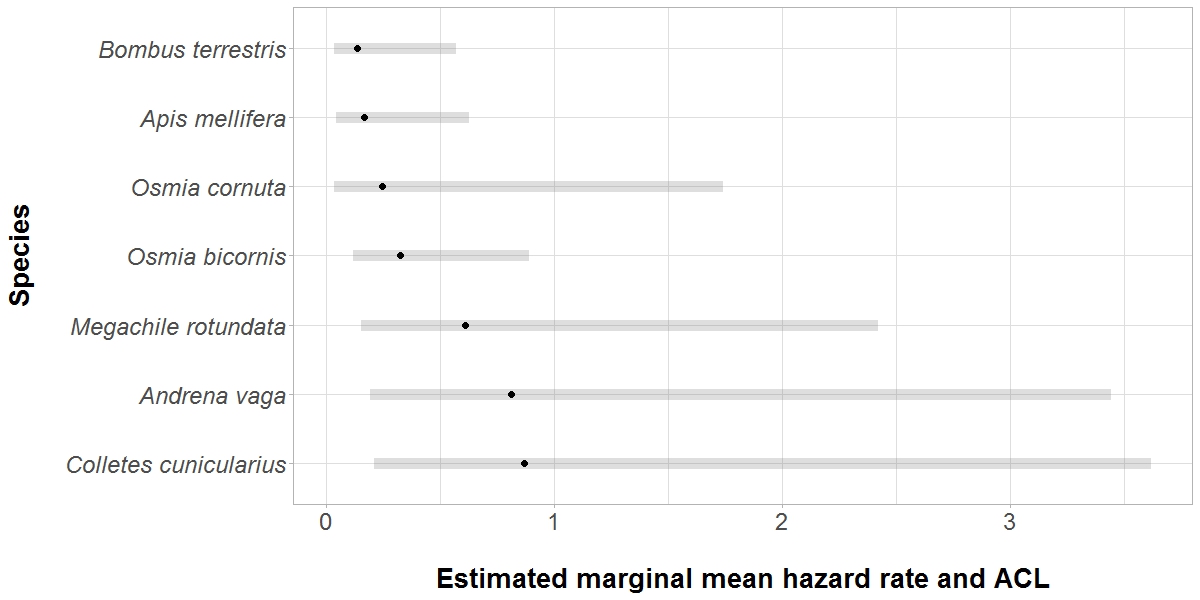


**(b)**


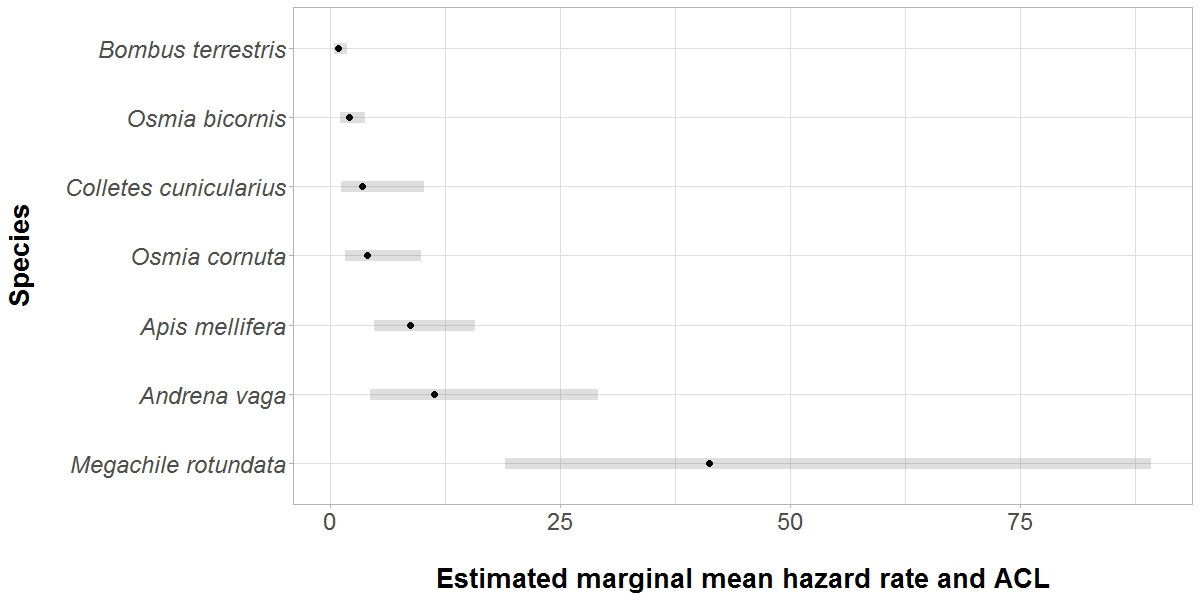


**Supplement 8**. Mean survival of bees per cage (after 96 hours) in the control (C) or the lambda-cyhalothrin (T) treatment in relation to mean bee weight per cage (in g). Each data point represents one cage; points were jittered for visibility. Bee species identity is represented by different colors. Survival (solid lines) ±asymptotic confidence intervals (shaded ribbons) were estimated via a Generalized Additive Mixed Model (gamm). The average weight of a bee individual was positively related to the probability of survival after 96 hours in the treatment group but not in the control crop (cf. **Supplement 9**). Mean ±standard error weight: *A. vaga* 125±1.8 mg, *B. terrestris* 253±4.1 mg, *C. cunicularius* 129±2.6 mg, *M. rotundata* 40±0.5 mg, *O. bicornis* 146±0.7mg, *O. cornuta* 159±4.4 mg.


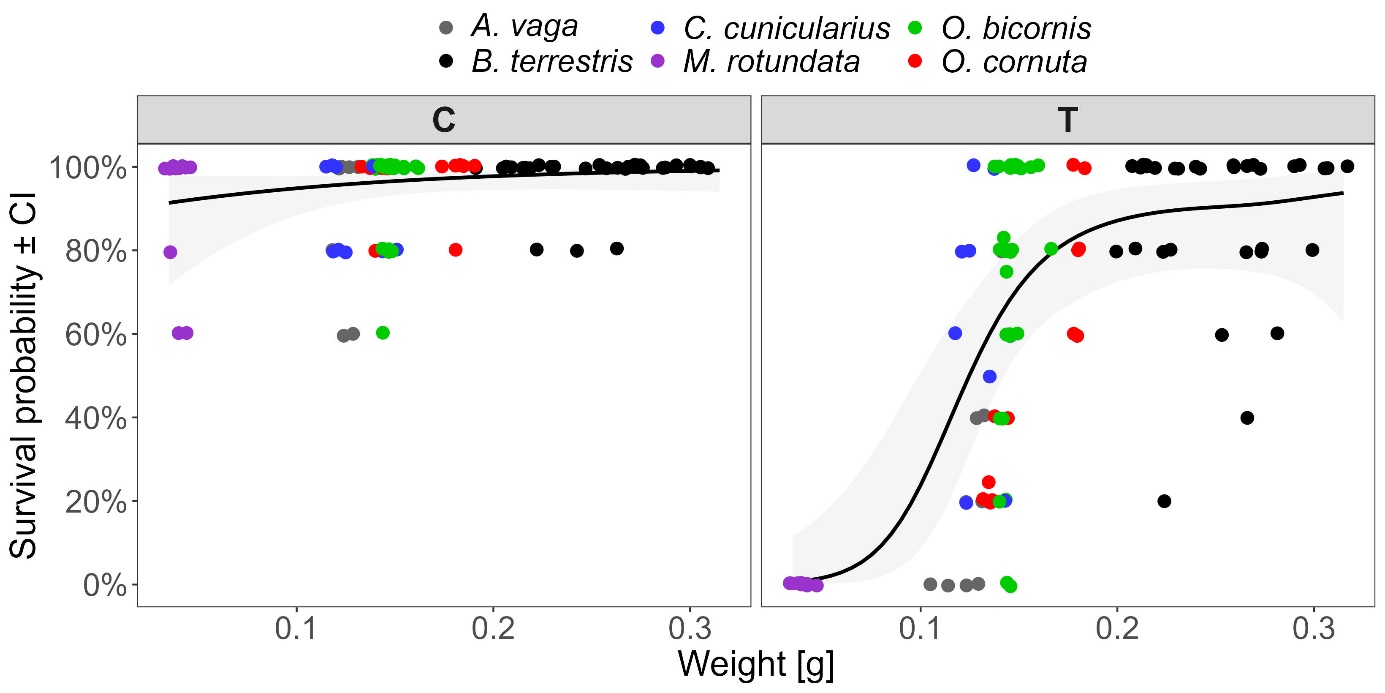


**Supplement 9**. Output of the Generalized Additive Mixed Model using a binomial distibution and containing mean *survival* of control and lambda-cyhalothrin-treated bees per cage (after 96 hours) as response variable, mean *weight* of a bee from that cage as a smoothed predictor variable, *treatment* (two levels) and its interaction with weight as a fixed factor, and *species* (six levels) as well as *trial number* as random variables. We used Akaike information criterion (AIC) for model selection between GLMM with and without quadratic terms and GAMM as well as for selection of the parameters in the final model. Analyses were conducted in R using the packages glmmTMB (version 1.1.7^5^) and gamm4 (version 0.2-6^6^). Model diagnostics were conducted using the R-package DHARMa (version 0.4.6^7^). Posthoc comparisons between control and lambda-cyhalothrin treatment were conducted with the R package emmeans (version 1.8.2^8^) on the log odds ratio scale at the 5^th^, 50^th^ and 95^th^ percentiles of the mean weight. Significant results at an alpha=0.05 level are indicated in bold and show that survival of treated bees is positively related to their weight in a non-linear way, while survival of control bees is not significantly related to their weight

| **Parametric coefficients** | **Estimate** | **Standard Error** | | **Z** | **P** |
| --- | --- | --- | --- | --- | --- |
| Intercept | 3.4712 | 0.3355 | | 10.345 | **<0.001** |
| Treatment T | -2.9461 | 0.2963 | | -9.943 | **<0.001** |
|  |  |  | |  |  |
| **Smooth terms** | **edf** | **Reference df** | | **Chi^2** | **P** |
| s(Weight) : Treatment C | 1.000 | 1.000 | | 3.023 | 0.082 |
| s(Weight) : Treatment T | 2.695 | 2.695 | | 27.553 | **<0.001** |
| Adjusted R^2=0.63 |  | |  |  |  |

| **Pairwise contrasts C – T** | | | | | |
| --- | --- | --- | --- | --- | --- |
| **Weight [g]** | **Odds ratio** | **Standard Error** | **df** | **t** | **P** |
| 0.040 | 1292.43 | 1592.18 | 210 | 5.816 | **<0.001** |
| 0.145 | 12.70 | 3.62 | 210 | 8.920 | **<0.001** |
| 0.293 | 7.97 | 6.79 | 210 | 2.435 | **0.016** |

**Supplement 10.** Model comparisons of full and alternative models for the behavioral parameters ‘symptoms’, ‘moribund’, ‘cramps’ and ‘affected’ after lambda-cyhalothrin application. Binomial generalized linear (mixed) models were fit with ML. Akaike information criterion (AIC) and Log-Likelihood (logLik) were used for model comparison. Models with the lowest AIC were considered as the final model (marked in bold). “^” indicates quadratic/cubic terms. “zi” indicates an addition of zero-inflation term. df=degree of freedom.

| **Behavior** | **Models** | **df** | **AIC** | **logLik** |
| --- | --- | --- | --- | --- |
| **Symptoms** | Random component |  |  |  |
|  | **~species * time + (time \| trial #/cage)** | **20** | **1189.0** | **-574.48** |
|  | ~species * time + (1 \| trial #/cage) | 16 | 1236.9 | -602.45 |
|  | ~species * time + (1 \| cage) | 15 | 1264.4 | -617.22 |
|  | ~species * time + (1 \| trial #) | 15 | 1429.5 | -699.72 |
|  | ~species * time | 14 | 1655.2 | -813.58 |
|  | ~species * time + time^2 + (1 \| trial #/cage) | 17 | 1235.4 | -600.71 |
|  | ~species * time + time^2 + time^3 + (1 \| trial #/cage) | 18 | 1231.2 | -597.60 |
|  | Fixed component |  |  |  |
|  | **~species * time + (time \| trial #/cage)** | **20** | **1189.0** | **-574.48** |
|  | ~species + time + (time \| trial #/cage) | 14 | 1262.3 | -617.17 |
| **Moribund** | Random component |  |  |  |
|  | ~species * time + (time \| trial #/cage) | 20 | 1091.3 | -525.66 |
|  | ~species * time + (1 \| trial #/cage) | 16 | 1184.8 | -577.40 |
|  | ~species * time + (1 \| cage) | 15 | 1202.5 | -586.28 |
|  | ~species * time + (1 \| trial #) | 15 | 1196.2 | -570.08 |
|  | ~species * time | 14 | 1281.0 | -626.50 |
|  | ~species * time + time^2 + (1 \| trial #/cage) | 17 | 1168.6 | -567.28 |
|  | ~species * time + time^2 + time^3 + (1 \| trial #/cage) | 18 | 1161.3 | -562.64 |
|  | **~species * time + (1 \| trial #/cage), zi=~species * time** | **30** | **1047.1** | **-493.54** |
|  | ~species * time + (1 \| cage), zi=~species * time | 29 | 1061.5 | -501.76 |
|  | ~species * time + (1 \| trial #), zi=~species * time | 29 | 1069.9 | -505.94 |
|  | ~species * time, zi=~species * time | 28 | 1096.1 | -520.06 |
|  | Fixed component |  |  |  |
|  | **~species * time + (1 \| trial #/cage), zi=~species * time** | **30** | **1047.1** | **-493.54** |
|  | ~species + time + (1 \| trial #/cage), zi=~species + time | 18 | 1053.1 | -508.55 |
| **Cramps** | Random component |  |  |  |
|  | **~species * time + (time \| trial #/cage)** | **20** | **1344.5** | **-652.25** |
|  | ~species * time + (1 \| trial #/cage) | 16 | 1364.4 | -727.19 |
|  | ~species * time + (1 \| cage) | 15 | 1377.7 | -673.84 |
|  | ~species * time + (1 \| trial #) | 15 | 1484.4 | -727.19 |
|  | ~species * time | 14 | 1683.3 | -827.63 |
|  | ~species * time + time^2 + (1 \| trial #/cage) | 17 | 1365.0 | -665.53 |
|  | ~species * time + time^2 + time^3 + (1 \| trial #/cage) | 18 | 1366.5 | -665.25 |
|  | Fixed component |  |  |  |
|  | **~species * time + (time \| trial #/cage)** | **20** | **1344.5** | **-652.25** |
|  | ~species + time + (1 \| trial #/cage) | 14 | 1446.5 | -709.25 |
| **Affected** | Random component |  |  |  |
|  | ~species * time + (1 \| trial #/cage) | 16 | 1008.8 | -488.41 |
|  | ~species * time + (1 \| cage) | 15 | 1031.9 | -500.96 |
|  | ~species * time + (1 \| trial #) | 15 | 1041.8 | -505.88 |
|  | ~species * time | 14 | 1110.3 | -541.14 |
|  | ~species * time + time^2 + (1 \| trial #/cage) | 17 | 851.5 | -408.73 |
|  | **~species * time + time^2 + time^3 + (1 \| trial #/cage)** | **18** | **841.4** | **-402.71** |
|  | ~species * time + time^2 + time^3 + (1 \| cage) | 17 | 864.0 | -415.02 |
|  | ~species * time + time^2 + time^3 + (1 \| trial #) | 17 | 883.5 | -424.76 |
|  | ~species * time + time^2 + time^3 | 16 | 952.3 | -460.14 |
|  | Fixed component |  |  |  |
|  | **~species * time + time^2 + time^3 + (1 \| trial #/cage)** | **18** | **841.4** | **-402.71** |
|  | ~species + time + time^2 + time^3 + (1 \| trial #/cage) | 12 | 968.5 | -472.27 |

**Supplement 11**. Species-specific probabilities of observing behavioral abnormalities after lambda-cyhalothrin application. Estimates of mean probability from linear models (cf. **Supplement 10**) were compared among all species (seven levels) for four sampling times (2, 24, 48 and 96 hours) after application. Post hoc tests were performed on the log odds ratio scale with a confidence level of 0.95 using multivariate t distribution adjustment (mvt) to account for multiple testing. Shown results are back-transformed to the response scale. If two or more means within a sampling time share the same grouping letter, they do not sinificantly differ from each other at a significance level of alpha=0.05. Results for the zero-inflated part were only included for the sampling time where any significant differences between species were detected. SE=standard error, LCL=asymptotic lower confidence level, UCL=asymptotic upper confidence level, co=conditional part of zero-inflated model, zi=zero-inflated part of zero-inflated model.

| Behavior | Time | Species | Probability | SE | LCL | UCL | Group | | | |
| --- | --- | --- | --- | --- | --- | --- | --- | --- | --- | --- |
| Symptoms | 2 | *A. mellifera* | 0.9885 | 0.0090 | 0.9158 | 0.9985 | a |  |  |  |
|  |  | *A. vaga* | 0.9990 | 0.0018 | 0.9229 | 1.0000 | a |  |  |  |
|  |  | *B. terrestris* | 0.2428 | 0.1269 | 0.0503 | 0.6601 |  | b |  |  |
|  |  | *C. cunicularius* | 0.8524 | 0.1153 | 0.3456 | 0.9844 |  |  | c |  |
|  |  | *M. rotundata* | 0.7356 | 0.1814 | 0.1960 | 0.9695 |  | b | c |  |
|  |  | *O. bicornis* | 0.9940 | 0.0045 | 0.9586 | 0.9992 | a |  |  |  |
|  |  | *O. cornuta* | 0.9651 | 0.0305 | 0.7220 | 0.9966 | a | c |  |  |
|  | 24 | *A. mellifera* | 0.6033 | 0.1574 | 0.2123 | 0.8956 | a | b |  |  |
|  |  | *A. vaga* | 0.9735 | 0.0299 | 0.6348 | 0.9987 | a |  | c |  |
|  |  | *B. terrestris* | 0.0849 | 0.0468 | 0.0187 | 0.3118 |  |  |  | d |
|  |  | *C. cunicularius* | 0.1586 | 0.1126 | 0.0200 | 0.6347 |  | b |  | d |
|  |  | *M. rotundata* | 1.0000 | 0.0000 | 0.9998 | 1.0000 |  |  |  | e |
|  |  | *O. bicornis* | 0.9481 | 0.0304 | 0.7823 | 0.9894 |  |  | c |  |
|  |  | *O. cornuta* | 0.8162 | 0.1153 | 0.3699 | 0.9711 | a |  | c |  |
|  | 48 | *A. mellifera* | 0.0182 | 0.0146 | 0.0022 | 0.1333 | a |  |  |  |
|  |  | *A. vaga* | 0.5126 | 0.2657 | 0.0621 | 0.9435 |  | b |  |  |
|  |  | *B. terrestris* | 0.0234 | 0.0148 | 0.0045 | 0.1137 | a |  |  |  |
|  |  | *C. cunicularius* | 0.0045 | 0.0052 | 0.0002 | 0.0854 | a |  |  |  |
|  |  | *O. bicornis* | 0.6207 | 0.1393 | 0.2601 | 0.8839 |  | b |  |  |
|  |  | *O. cornuta* | 0.3767 | 0.1957 | 0.0647 | 0.8407 |  | b |  |  |
|  | 96 | *A. mellifera* | 0.0000 | 0.0000 | 0.0000 | 0.0002 | a |  |  |  |
|  |  | *A. vaga* | 0.0009 | 0.0019 | 0.0000 | 0.2376 | a | b |  |  |
|  |  | *B. terrestris* | 0.0016 | 0.0017 | 0.0001 | 0.0236 |  | b |  |  |
|  |  | *C. cunicularius* | 0.0000 | 0.0000 | 0.0000 | 0.0010 | a |  |  |  |
|  |  | *O. bicornis* | 0.0129 | 0.0117 | 0.0012 | 0.1255 |  | b |  |  |
|  |  | *O. cornuta* | 0.0111 | 0.0155 | 0.0003 | 0.3143 |  | b |  |  |
| Moribund | 2  (co) | *A. mellifera* | 0.1653 | 0.1434 | 0.0123 | 0.7585 | a | b |  |  |
|  |  | *A. vaga* | 0.2175 | 0.1038 | 0.0521 | 0.5845 | a | b |  |  |
|  |  | *B. terrestris* | 0.1378 | 0.0759 | 0.0284 | 0.4662 | a |  |  |  |
|  |  | *C. cunicularius* | 0.2064 | 0.1233 | 0.0339 | 0.6581 | a | b |  |  |
|  |  | *M. rotundata* | 0.7345 | 0.1550 | 0.2500 | 0.9581 |  | b |  |  |
|  |  | *O. bicornis* | 0.4496 | 0.1152 | 0.1920 | 0.7380 | a | b |  |  |
|  |  | *O. cornuta* | 0.3691 | 0.1367 | 0.1090 | 0.7359 | a | b |  |  |
|  | 2 | *A. mellifera* | 2.6290 | 0.8330 | 0.3950 | 4.8630 | a |  |  |  |
|  | (zi) | *A. vaga* | -12.029 | 165.10 | -454.98 | 430.92 | a | b |  |  |
|  |  | *B. terrestris* | 1.8102 | 0.5380 | 0.3660 | 3.2550 | a |  |  |  |
|  |  | *C. cunicularius* | -0.9935 | 1.1910 | -4.1880 | 2.2010 | a | b |  |  |
|  |  | *M. rotundata* | 0.8238 | 0.6980 | -1.0500 | 2.6970 | a | b |  |  |
|  |  | *O. bicornis* | 0.0163 | 0.2780 | -0.7290 | 0.7610 |  | b |  |  |
|  |  | *O. cornuta* | 0.3563 | 0.5030 | -0.9930 | 1.7060 | a | b |  |  |
|  | 24 | *A. mellifera* | 0.0469 | 0.0349 | 0.0062 | 0.2801 | a |  |  |  |
|  |  | *A. vaga* | 0.1644 | 0.0826 | 0.0384 | 0.4919 | a | b |  |  |
|  |  | *B. terrestris* | 0.0440 | 0.0223 | 0.0112 | 0.1577 | a |  |  |  |
|  |  | *C. cunicularius* | 0.0651 | 0.0612 | 0.0048 | 0.4998 | a | b |  |  |
|  |  | *M. rotundata* | 0.5149 | 0.2004 | 0.1120 | 0.8989 |  | b |  |  |
|  |  | *O. bicornis* | 0.3169 | 0.0924 | 0.1300 | 0.5899 |  | b |  |  |
|  |  | *O. cornuta* | 0.1604 | 0.0717 | 0.0445 | 0.4392 | a | b |  |  |
|  | 48 | *A. mellifera* | 0.0107 | 0.0087 | 0.0012 | 0.0852 | a | b |  |  |
|  |  | *A. vaga* | 0.1189 | 0.0734 | 0.0213 | 0.4559 |  | b | c |  |
|  |  | *B. terrestris* | 0.0117 | 0.0067 | 0.0026 | 0.0506 | a |  |  |  |
|  |  | *C. cunicularius* | 0.0163 | 0.0255 | 0.0003 | 0.5115 | a | b | c |  |
|  |  | *O. bicornis* | 0.2002 | 0.0713 | 0.0727 | 0.4440 |  |  | c |  |
|  |  | *O. cornuta* | 0.0533 | 0.0294 | 0.0122 | 0.2041 | a | b |  |  |
|  | 96 | *A. mellifera* | 0.0005 | 0.0008 | 0.0000 | 0.0369 | a | b |  |  |
|  |  | *A. vaga* | 0.0597 | 0.0613 | 0.0036 | 0.5278 | a |  | c |  |
|  |  | *B. terrestris* | 0.0008 | 0.0008 | 0.0001 | 0.0113 |  | b |  |  |
|  |  | *C. cunicularius* | 0.0009 | 0.0028 | 0.0000 | 0.6974 | a | b | c |  |
|  |  | *O. bicornis* | 0.0679 | 0.0399 | 0.0137 | 0.2765 |  |  | c |  |
|  |  | *O. cornuta* | 0.0049 | 0.0044 | 0.0004 | 0.0508 | a | b | c |  |
| Cramps | 2 | *A. mellifera* | 0.9848 | 0.0105 | 0.9110 | 0.9976 | a |  |  |  |
|  |  | *A. vaga* | 0.6434 | 0.1892 | 0.1710 | 0.9403 |  | b | c |  |
|  |  | *B. terrestris* | 0.1285 | 0.0651 | 0.0311 | 0.4042 |  |  |  | d |
|  |  | *C. cunicularius* | 0.1132 | 0.0822 | 0.0146 | 0.5233 |  |  |  | d |
|  |  | *M. rotundata* | 0.3090 | 0.1650 | 0.0555 | 0.7730 |  | b |  | d |
|  |  | *O. bicornis* | 0.9029 | 0.0501 | 0.6750 | 0.9766 |  |  | c |  |
|  |  | *O. cornuta* | 0.6850 | 0.1568 | 0.2440 | 0.9362 |  | b | c |  |
|  | 24 | *A. mellifera* | 0.1033 | 0.0542 | 0.0239 | 0.3514 | a | b |  |  |
|  |  | *A. vaga* | 0.3994 | 0.1621 | 0.1000 | 0.7989 | a |  | c |  |
|  |  | *B. terrestris* | 0.0384 | 0.0180 | 0.0109 | 0.1266 |  | b |  |  |
|  |  | *C. cunicularius* | 0.0451 | 0.0308 | 0.0071 | 0.2379 |  | b |  |  |
|  |  | *M. rotundata* | 0.5869 | 0.2052 | 0.1320 | 0.9302 | a |  | c |  |
|  |  | *O. bicornis* | 0.6813 | 0.0963 | 0.3980 | 0.8735 |  |  | c |  |
|  |  | *O. cornuta* | 0.3815 | 0.1434 | 0.1100 | 0.7548 | a |  | c |  |
|  | 48 | *A. mellifera* | 0.0001 | 0.0001 | 0.0000 | 0.0016 | a |  |  |  |
|  |  | *A. vaga* | 0.1828 | 0.1057 | 0.0339 | 0.5881 |  | b |  |  |
|  |  | *B. terrestris* | 0.0095 | 0.0052 | 0.0023 | 0.0392 |  |  | c |  |
|  |  | *C. cunicularius* | 0.0157 | 0.0133 | 0.0017 | 0.1312 |  |  | c | d |
|  |  | *O. bicornis* | 0.3006 | 0.0756 | 0.1430 | 0.5245 |  | b |  |  |
|  |  | *O. cornuta* | 0.1350 | 0.0739 | 0.0289 | 0.4506 |  | b |  | d |
|  | 96 | *A. mellifera* | <0.0001 | <0.0001 | <0.0001 | <0.0001 | a |  |  |  |
|  |  | *A. vaga* | 0.0247 | 0.0294 | 0.0010 | 0.3836 |  | b | c |  |
|  |  | *B. terrestris* | 0.0006 | 0.0006 | 0.0000 | 0.0075 |  | b |  |  |
|  |  | *C. cunicularius* | 0.0018 | 0.0028 | 0.0000 | 0.0993 |  | b | c |  |
|  |  | *O. bicornis* | 0.0171 | 0.0080 | 0.0050 | 0.0571 |  |  | c |  |
|  |  | *O. cornuta* | 0.0099 | 0.0103 | 0.0006 | 0.1363 |  | b | c |  |
| Affected | 2 | *A. mellifera* | 0.0190 | 0.0099 | 0.0047 | 0.0731 | a | b |  |  |
|  |  | *A. vaga* | 0.0002 | 0.0003 | 0.0000 | 0.0246 | a |  | c |  |
|  |  | *B. terrestris* | 0.0215 | 0.0106 | 0.0058 | 0.0768 | a | b |  |  |
|  |  | *C. cunicularius* | 0.3310 | 0.1340 | 0.0903 | 0.7110 |  |  |  | d |
|  |  | *M. rotundata* | 0.0523 | 0.0338 | 0.0089 | 0.2520 |  | b |  | d |
|  |  | *O. bicornis* | 0.0006 | 0.0005 | 0.0001 | 0.0045 |  |  | c |  |
|  |  | *O. cornuta* | 0.0054 | 0.0040 | 0.0008 | 0.0376 | a | b | c |  |
|  | 24 | *A. mellifera* | 0.1750 | 0.0701 | 0.0556 | 0.4350 | a |  |  |  |
|  |  | *A. vaga* | 0.0133 | 0.0163 | 0.0005 | 0.2690 | a | b |  |  |
|  |  | *B. terrestris* | 0.1080 | 0.0444 | 0.0343 | 0.2910 | a |  |  |  |
|  |  | *C. cunicularius* | 0.0235 | 0.0254 | 0.0013 | 0.3110 | a | b |  |  |
|  |  | *M. rotundata* | 0.1820 | 0.1320 | 0.0206 | 0.7010 | a | b |  |  |
|  |  | *O. bicornis* | 0.0284 | 0.0147 | 0.0071 | 0.1070 |  | b |  |  |
|  |  | *O. cornuta* | 0.1360 | 0.0704 | 0.0309 | 0.4360 | a | b |  |  |
|  | 48 | *A. mellifera* | 0.0821 | 0.0409 | 0.0213 | 0.2688 | a | b |  |  |
|  |  | *A. vaga* | 0.0449 | 0.0410 | 0.0039 | 0.3627 | a | b |  |  |
|  |  | *B. terrestris* | 0.0233 | 0.0124 | 0.0057 | 0.0902 | a |  |  |  |
|  |  | *C. cunicularius* | <0.0001 | 0.0001 | <0.0001 | 0.0081 |  |  | c |  |
|  |  | *O. bicornis* | 0.0587 | 0.0270 | 0.0172 | 0.1819 | a | b |  |  |
|  |  | *O. cornuta* | 0.1590 | 0.0797 | 0.0382 | 0.4718 |  | b |  |  |
|  | 96 | *A. mellifera* | 0.0003 | 0.0004 | <0.0001 | 0.0080 | a | b |  |  |
|  |  | *A. vaga* | 0.0102 | 0.0197 | 0.0001 | 0.6230 | a |  |  |  |
|  |  | *B. terrestris* | <0.0001 | <0.0001 | <0.0001 | 0.0006 |  | b |  |  |
|  |  | *C. cunicularius* | <0.0001 | <0.0001 | <0.0001 | <0.0001 |  |  | c |  |
|  |  | *O. bicornis* | 0.0051 | 0.0041 | 0.0006 | 0.0408 | a |  |  |  |
|  |  | *O. cornuta* | 0.0049 | 0.0054 | 0.0003 | 0.0807 | a |  |  |  |

**Supplement 12**. Species-specific probabilities of observing behavioral abnormalities after lambda-cyhalothrin application. Solid lines show model estimates from linear models, dotted lines represent asymptotic confidence intervals at a confidence level of 0.95. Filled circles depict jittered raw data for each cage in each trial. For the parameter ‘moribund’, plots are shown for the conditional part (B) and the zero-inflated part (C) of the model.


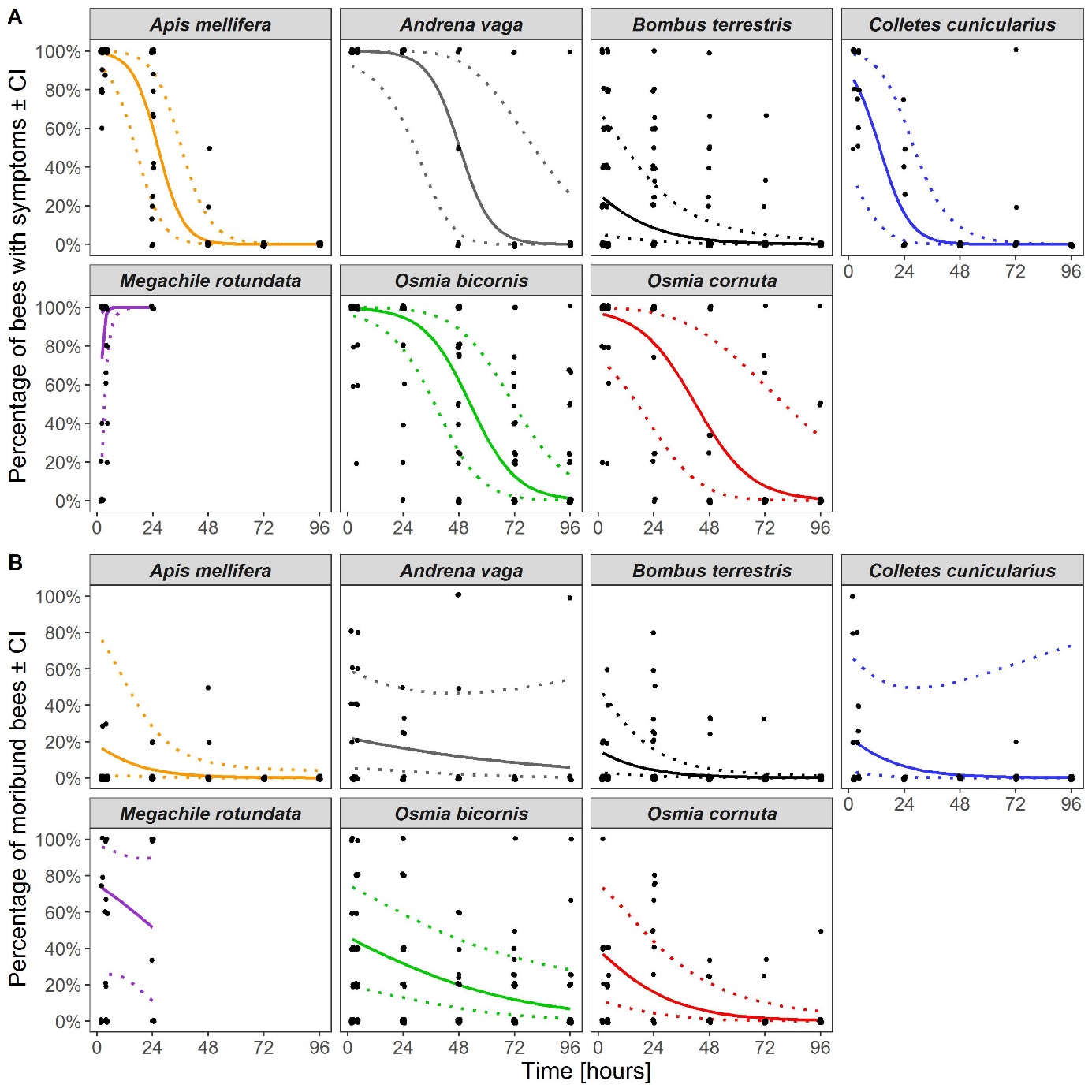


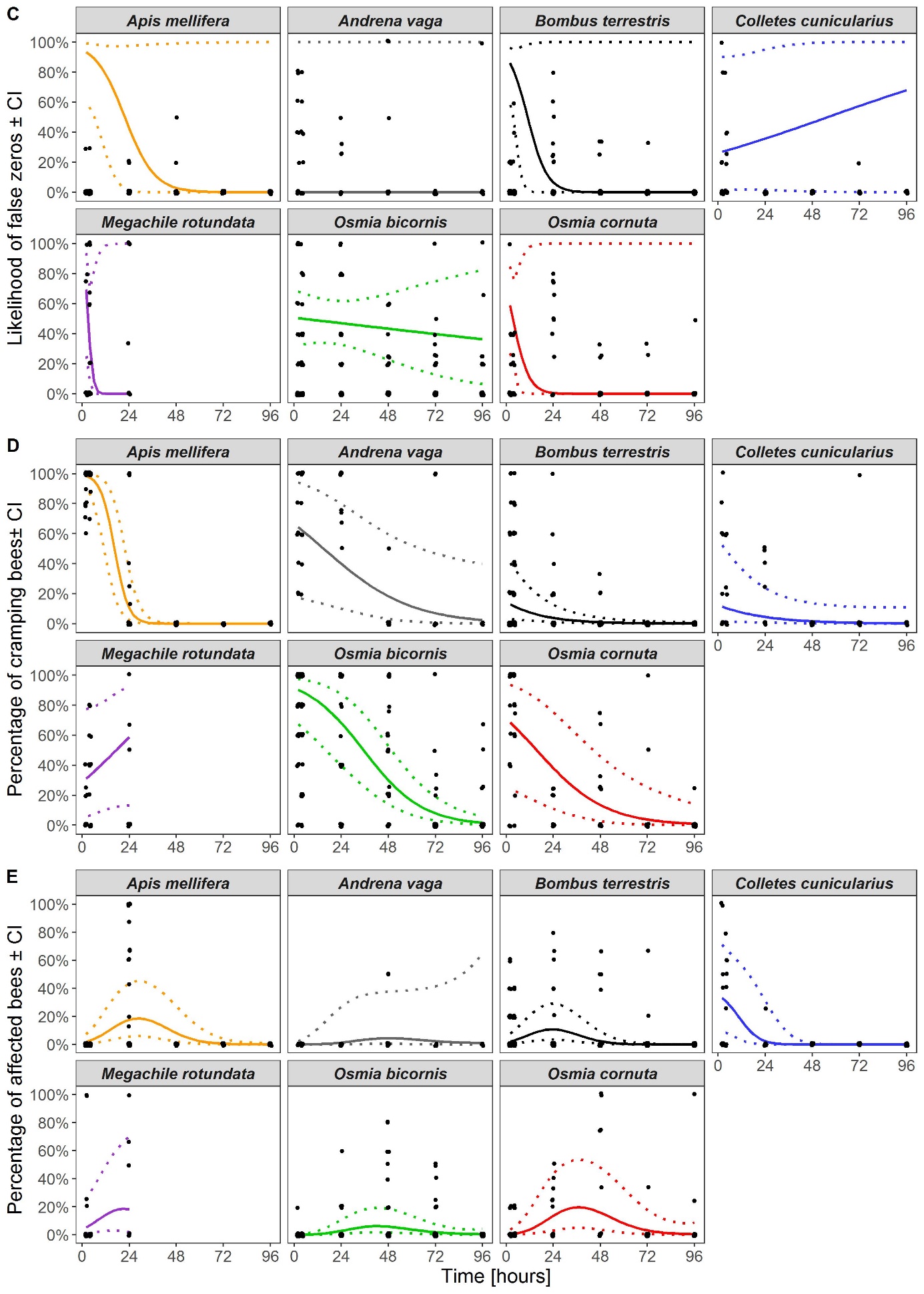


References

1. Thompson, H. M. & Pamminger, T. Are honeybees suitable surrogates for use in pesticide risk assessment for non-*Apis* bees? *Pest Manag. Sci.* **75,** 2549–2557; 10.1002/ps.5494 (2019).

2. OECD. *Test No. 214: Honeybees, acute contact toxicity test.* OECD Guidelines for the Testing of Chemicals (Organisation for Economic Co-operation and Development, 1998).

3. Seeley, T. D. & Kolmes, S. A. Age Polyethism for Hive Duties in Honey Bees — Illusion or Reality? *Ethology* **87,** 284–297; 10.1111/j.1439-0310.1991.tb00253.x (1991).

4. Goerzen, D. W. Alfalfa leafcutting bee incubation calendar. Available at <https://saspa.com/PDF/Alfalfa%20leafcutting%20bee%20incubation%20calendar%20-%20May%202022.pdf> (updated version) (2017).

5. Brooks, M. E. *et al.* glmmTMB balances speed and flexibility among packages for zero-inflated Generalized Linear Mixed Modeling. *The R Journal* **9,** 378–400; 10.32614/RJ-2017-066 (2017).

6. Wood, S. & Scheipl, F. *gamm4: Generalized Additive Mixed Models using ‘mgcv’ and ‘lme4’. R package version 0.2-6,* [*https://CRAN.R-project.org/package=gamm4*](https://CRAN.R-project.org/package=gamm4) (2020).

7. Hartig, F. *DHARMa: Residual Diagnostics for Hierarchical (Multi-Level / Mixed) Regression Models. R package version 0.4.6,* [*https://CRAN.R-project.org/package=DHARMa*](https://CRAN.R-project.org/package=DHARMa) (2022).

8. Lenth, R. *emmeans: Estimated Marginal Means, aka Least-Squares Means. R package version 1.8.2,* [*https://CRAN.R-project.org/package=emmeans*](https://CRAN.R-project.org/package=emmeans) (2022).
